# Single copy/knock-in models of ALS SOD1 in *C*. *elegans* suggest loss and gain of function have different contributions to cholinergic and glutamatergic neurodegeneration

**DOI:** 10.1371/journal.pgen.1007682

**Published:** 2018-10-08

**Authors:** Saba N. Baskoylu, Jill Yersak, Patrick O’Hern, Sarah Grosser, Jonah Simon, Sarah Kim, Kelsey Schuch, Maria Dimitriadi, Katherine S. Yanagi, Jeremy Lins, Anne C. Hart

**Affiliations:** 1 Department of Neuroscience and Carney Institute for Brain Sciences, Brown University, Providence, Rhode Island, United States of America; 2 Department of Molecular Biology, Cellular Biology & Biochemistry, Brown University, Providence, Rhode Island, United States of America; Stanford University School of Medicine, UNITED STATES

## Abstract

Mutations in Cu/Zn superoxide dismutase 1 (SOD1) lead to Amyotrophic Lateral Sclerosis (ALS), a neurodegenerative disease that disproportionately affects glutamatergic and cholinergic motor neurons. Previous work with SOD1 overexpression models supports a role for SOD1 toxic gain of function in ALS pathogenesis. However, the impact of SOD1 loss of function in ALS cannot be directly examined in overexpression models. In addition, overexpression may obscure the contribution of SOD1 loss of function in the degeneration of different neuronal populations. Here, we report the first single-copy, ALS knock-in models in *C*. *elegans* generated by transposon- or CRISPR/Cas9- mediated genome editing of the endogenous *sod-1* gene. Introduction of ALS patient amino acid changes A4V, H71Y, L84V, G85R or G93A into the *C*. *elegans sod-1* gene yielded single-copy/knock-in ALS SOD1 models. These differ from previously reported overexpression models in multiple assays. In single-copy/knock-in models, we observed differential impact of *sod-1* ALS alleles on glutamatergic and cholinergic neurodegeneration. A4V, H71Y, G85R, and G93A animals showed increased SOD1 protein accumulation and oxidative stress induced degeneration, consistent with a toxic gain of function in cholinergic motor neurons. By contrast, H71Y, L84V, and G85R lead to glutamatergic neuron degeneration due to *sod-1* loss of function after oxidative stress. However, dopaminergic and serotonergic neuronal populations were spared in single-copy ALS models, suggesting a neuronal-subtype specificity previously not reported in invertebrate ALS SOD1 models. Combined, these results suggest that knock-in models may reproduce the neurotransmitter-type specificity of ALS and that both SOD1 loss and gain of toxic function differentially contribute to ALS pathogenesis in different neuronal populations.

## Author summary

In all SOD1 ALS patients, cholinergic spinal motor neurons degenerate, but degeneration of cortical glutamatergic neurons is less common. Despite decades of work, it remains unclear why some disease alleles (*e*.*g*. A4V) primarily affect cholinergic spinal neurons, while other alleles affect both cholinergic and glutamatergic neurons. New genome editing techniques allowed us to create the first *C*. *elegans* knock-in/single-copy models for SOD1 ALS by directly editing the *C*. *elegans sod-1* gene to recreate SOD1 amino acid changes that cause ALS in patients. These new models are complementary to previously described overexpression models, which revealed mutant SOD1 toxic gain of function properties. By contrast, in the new *C*. *elegans* knock-in models, we find that both loss and gain of *sod-1* function contribute to neurodegeneration. *C*. *elegans* cholinergic motor neuron loss is primarily driven by toxic gain of function, but glutamatergic neuron loss is primarily driven by loss of function. Only cholinergic and glutamatergic neurons degenerate in *C*. *elegans* knock-in models; dopaminergic, serotoninergic and GABAergic neurons do not. This pattern of neuronal loss is reminiscent of the pattern of neuronal loss seen in SOD1 ALS patients. Strikingly, in the *C*. *elegans* A4V model, only cholinergic neurons are lost. Our results suggest that an underlying premise of the ALS field–that identical pathological mechanisms lead to degeneration of cholinergic and glutamatergic neurons–should be reconsidered. Mechanisms that predominantly drive glutamatergic and cholinergic neuron degeneration in ALS may not be identical.

## Introduction

Amyotrophic lateral sclerosis (ALS) is an adult-onset fatal neurodegenerative disorder marked by the progressive loss of glutamatergic and cholinergic motor neurons. It is the most common motor neuron disorder affecting adults. The first ALS-linked mutations were discovered in a gene encoding the antioxidant enzyme Cu/Zn superoxide dismutase 1 (SOD1) [1]. Roughly 1% of all cases are caused by mutations of SOD1.

In patients, misfolded SOD1 is a major constituent of proteinaceous inclusions in the affected neurons [2–4] and pathogenic SOD1 variants inevitably lead to cholinergic motor neuron degeneration. However, ALS is inherently heterogeneous: relative involvement of the glutamatergic corticospinal tract [5] and glutamatergic sensory neurons [6,7] differs greatly among patients, and clinical presentation of ALS, including age of disease onset, progression, severity and duration, varies [8,9]. Consequently, how mutant SOD1 mediates its toxic function in different neuronal populations remains largely unknown.

Published studies to date have primarily relied on human mutant SOD1 protein overexpression models to examine neuronal dysfunction [10–15]. Overexpression models can recapitulate several key aspects of ALS pathogenesis, including motor neuron degeneration, protein aggregation and motor dysfunction. Furthermore, most ALS SOD1 alleles have a dominant pattern of inheritance [16]. These findings support a role for gain of toxic SOD1 function in disease pathogenesis.

However, SOD1 overexpression models may not permit the study of disease mechanisms in entirety for several reasons. First, overexpression of wild type SOD1 protein has deleterious effects in model organisms [17,18], making it difficult to dissociate the impact of ALS mutations from protein overexpression. Second, overexpression models make it challenging to discover SOD1 loss of function contributions to ALS pathogenesis. Recently, single-copy SOD1 knock-in models in flies, fish and mice have been reported [19–21]. Defects observed in these knock-in models differ dramatically from those observed in overexpression models and are often less severe. Furthermore, knock-in mice and fly models suggest that both SOD1 loss and gain of function may contribute to disease pathogenesis for the limited number of SOD1 alleles tested. A single-copy ALS SOD1 knock-in mouse model shows peripheral neuropathy, reminiscent of SOD1 null mice [20]. And, eclosion defects in single-copy ALS SOD1 knock-in models in flies were rescued by the introduction of wild type SOD1, consistent with a loss of function defect [19]. Still, why SOD1 patient alleles differ in presentation and progression remains unclear, and overexpression models likely complicate this analysis.

Previously reported ALS SOD1 overexpression models in *C*. *elegans* demonstrate neuronal and muscular dysfunction [13,22–24]. To compliment these overexpression models, we generated single-copy ALS SOD1 knock-in models in *C*. *elegans*. We edited the *C*. *elegans sod-1* gene to create A4V, H71Y, L84V, G85R and G93A missense mutations. In the resulting single-copy ALS model animals, we observed oxidative stress induced glutamatergic and cholinergic neuron degeneration. While H71Y and G85R affected both cholinergic and glutamatergic neurons, A4V and G93A models affected cholinergic neurons only. However, other neuron classes were relatively spared. Overall, we found that cholinergic and glutamatergic neurons are differentially sensitive to SOD-1 loss and gain of toxic function. Our results suggest that both loss and gain of toxic SOD-1 function may be involved in disease pathogenesis.

## Results

### Generation of single-copy ALS SOD1 knock-in models in *C*. *elegans*

**Fig 1:**
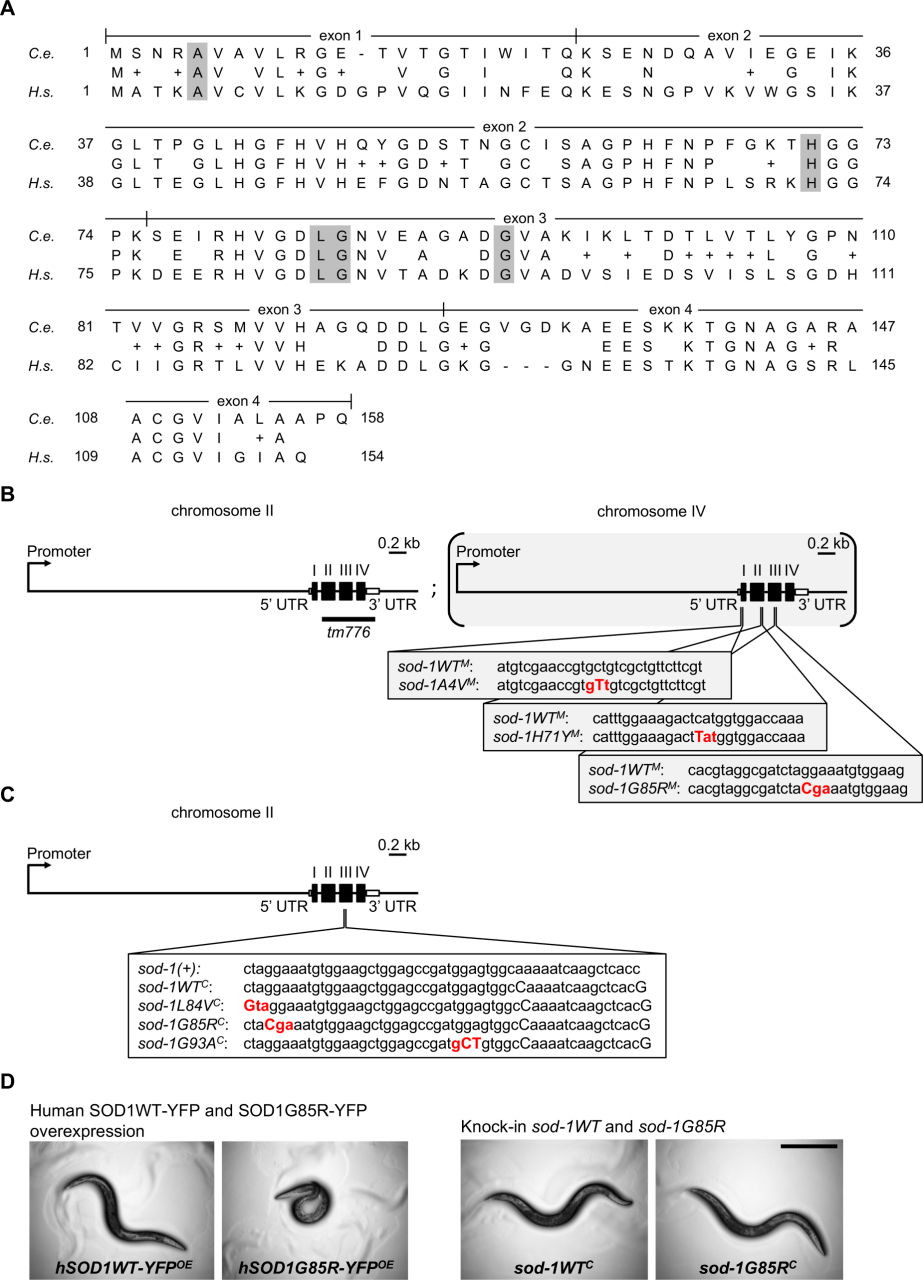
*C*. *elegans* model construction for ALS SOD1. **(A)** Alignment of the *C*. *elegans* SOD-1 and human SOD1 proteins. Conserved amino acid residues are listed between the protein sequences; similar amino acids are indicated with a “+”. Exon boundaries of *C*. *elegans sod-1* are indicated. Shaded residues correspond to those mutated herein to create *C*. *elegans* ALS SOD1 single-copy models for A4V, H71Y, L84V, G85R or G93A using strategies described in panels B and C. *C*.*e*. for *C*. *elegans* SOD-1 GenPept Accession Number CCD64618.; *H*.*s*. for *Homo sapiens* SOD1 GenPept Accession Number CAG46542. Exon/intron boundaries are from *C*. *elegans sod-1* transcript *C15F1*.*7b*.*1*. encoding isoform b. **(B)** Single-copy ALS *sod-1* models generated by Mos1-mediated Single Copy Insertion (MosSCI). A single copy of the entire *sod-1* gene containing an ALS SOD1 mutation (A4V, H71Y or G85R) was inserted at a targeted site on chromosome IV along with an *unc-119(+)* rescue construct (not illustrated). A wild type *sod-1WT*^*M*^ control with the *unc-119(+)* rescue construct and a negative “*empty*” control with the *unc-119(+)* rescue construct alone (not illustrated) were similarly integrated for rescue or to control for insertion site positional effects, respectively. Transgenes were crossed onto the *sod-1(tm776)* II null background to generate single-copy model animals homozygous for transgenes and *sod-1(tm776)*. All alleles generated by MosSCI are designated with an “M” superscript. **(C)** Single-copy ALS *sod-1* models generated by CRISPR/Cas9-mediated homologous recombination. The endogenous *sod-1* locus on chromosome II was edited using CRISPR/Cas9-mediated homologous recombination to generate mutations corresponding to L84V, G85R and G93A. A control wild type *sod-1WT*^*C*^ was also generated containing all the silent codon changes needed for CRISPR/Cas9 genome editing. All alleles generated by CRISPR/Cas9 are annotated with a “C” superscript. **(D)** *C*. *elegans* overexpression and single-copy models differ. Left pair of images: animals overexpressing human SOD1G85R-YFP in neurons [13] show pronounced posture/locomotion defects, when compared to animals overexpressing wild type human SOD1-YFP protein. Right pair: Single-copy ALS *sod-1* animals and controls had no discernible locomotion defects under normal culture conditions. Single-copy/knock-in *sod-1G85R*^*M*^ animals are shown; no locomotion defect was observed in more than 150 animals examined for each single-copy strain. *hSOD1WT-YFP*^*OE*^: neuronal human SOD1WT overexpression model. *hSOD1G85R-YFP*^*OE*^: neuronal human SOD1G85R-YFP overexpression model. Scale bar = 0.5 mm.

To design single-copy *C*. *elegans* ALS SOD1 models, we compared human SOD1 and *C*. *elegans* SOD-1 protein sequences. Alignment of the two proteins revealed 71% similarity and 56% identity between species (Fig 1A; P00441 and C15F1.7b.1, BLAST). To create A4V, H71Y, L84V, G85R and G93A models, we mutated conserved amino acid residues in the *C*. *elegans sod-1* gene, and generated single-copy ALS models using two different strategies (Fig 1B and 1C). To avoid confusion, *C*. *elegans* models generated herein were named based on human amino acid numbering. Formal allele designations with *C*. *elegans* amino acid changes can be found in S3 Table.

Using Mos1-mediated single copy insertion (MosSCI [25]), we recreated ALS SOD1 mutations for A4V, H71Y, and G85R. MosSCI relies on excision of a known transposon to facilitate insertion of transgenic DNA fragments into a previously defined genomic location. ALS-associated *sod-1* alleles for A4V, H71Y, and G85R were individually inserted into the MosSCI *cxTi10882* site on chromosome IV with the *sod-1* promoter, exons, introns and 3’ sequences (Fig 1B). To control for the *sod-1* gene relocalization, we also inserted the entire wild type *sod-1* gene in the same location on chromosome IV (Fig 1B). To facilitate the selection of transgenic animals, these transgenes were introduced alongside an *unc-119(+)* rescue construct. Therefore, an additional “empty” negative control carrying the *unc-119(+)* construct alone was generated; these lack any of the *sod-1* sequences inserted in the remainder of the MosSCI models. All alleles generated by MosSCI were named with an “M” superscript (*sod-1WT*^*M*^, *sod-1A4V*^*M*^, *sod-1H71Y*^*M*^, *sod-1G85R*^*M*^ *and empty*^*M*^), to distinguish them from the endogenous *C*. *elegans sod-1* gene on chromosome II. Subsequently, each transgene was crossed into the *sod-1(tm776)* loss of function background, referred to hereafter as *sod-1(-)*. The resulting strains were homozygous for control or single-copy ALS *sod-1* transgenes. All the comparisons between ALS *sod-1* models and controls reported here were made in *sod-1(-)* background, unless indicated otherwise.

Additionally, using CRISPR/Cas9-mediated homologous recombination (HR), we directly edited the endogenous *C*. *elegans sod-1* gene to recreate ALS SOD1 mutations for L84V, G85R and G93A in the endogenous *sod-1* gene on chromosome II (Fig 1C). CRISPR/Cas9-mediated genome editing requires introduction of silent codon changes into endogenous *sod-1*. Consequently, a wild type control was generated containing identical silent mutations (Fig 1C). Models generated by CRISPR/Cas9 were named with a “C” superscript (*sod-1WT*^*C*^, *sod-1L84V*^*C*^, *sod-1G85R*^*C*^ and *sod-1G93A*^*C*^). To demonstrate reproducibility across strains generated by MosSCI and CRISPR/Cas9, we created the G85R allele twice, once using each technique (Fig 1B and 1C).

Single-copy models may differ from overexpression models in severity and type of defects observed [19,20]. Thus, we compared the new single-copy ALS *sod-1* models to previously published neuronal overexpression models provided by the Horwich lab [13]. These animals overexpress human SOD1G85R protein in neurons and have severe locomotion defects (Fig 1D), while animals overexpressing wild type human SOD1 have relatively normal locomotion. We found that none of the single-copy ALS *sod-1* model animals carrying patient amino acid changes had overt locomotion defects (Fig 1D). Furthermore, lifespan was only slightly decreased in single-copy ALS *sod-1* animals, with the exception of G85R models, which had normal lifespan (S1A and S1B Fig).

### ALS *sod-1* alleles and oxidative stress accelerate the formation of human SOD1-YFP inclusions in *C*. *elegans* motor neurons

**Fig 2:**
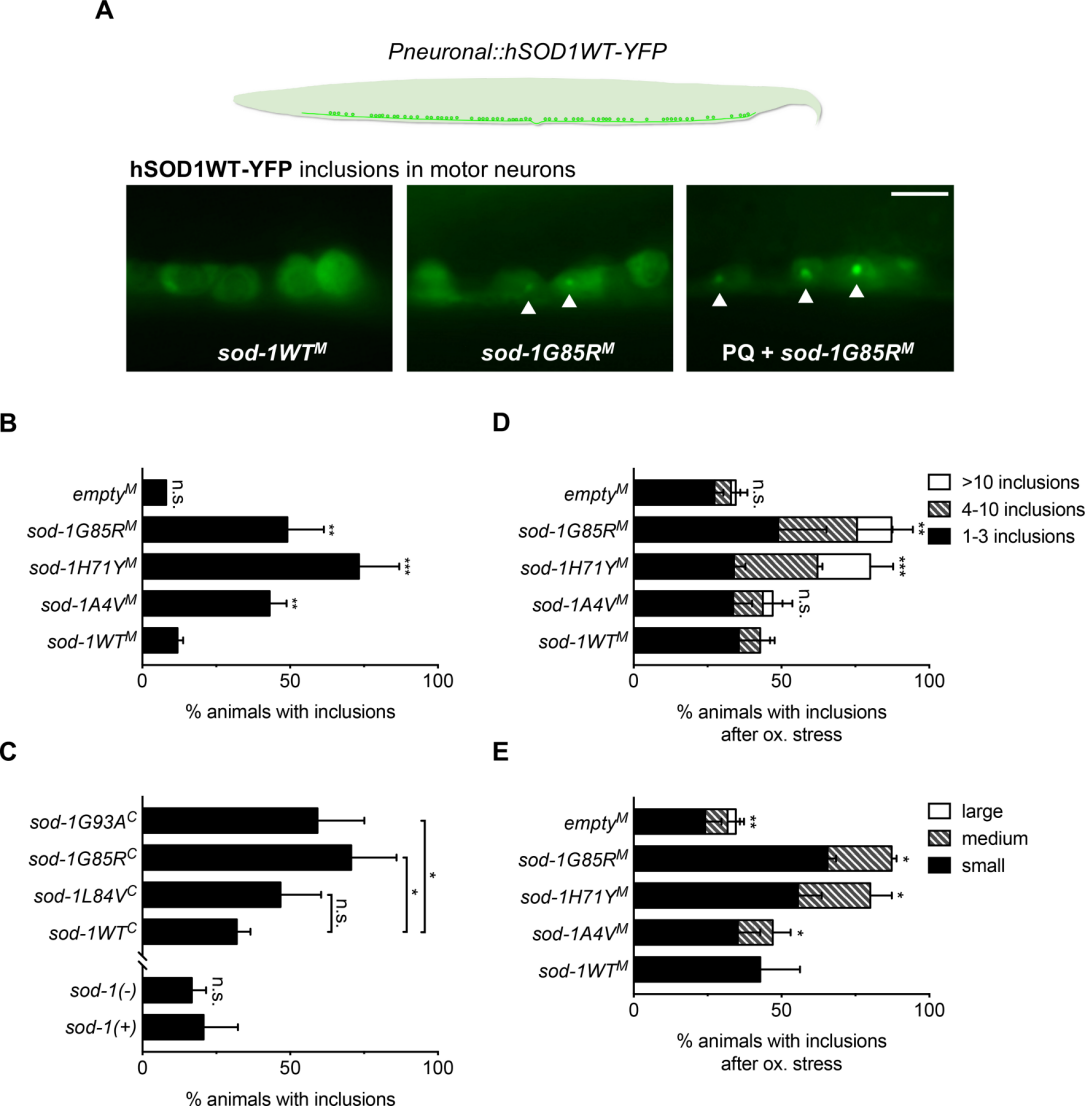
*C*. *elegans* single-copy/knock-in *sod-1* alleles increase SOD1 inclusions in motor neurons. **(A)** Top: Cartoon illustration of the motor neurons examined to score hSOD1WT-YFP inclusion formation. Bottom: representative images of *sod-1WT*^*M*^ and *sod-1G85R*^*M*^ animals expressing hSOD1WT-YFP fusion protein [13] in ventral nerve cord motor neurons. *sod-1G85R*^*M*^ animals had small neuronal inclusions that were largely absent in *sod-1WT*^*M*^ wild type controls. Paraquat-induced oxidative stress (PQ or ox.) increased the number and size of the inclusions in *sod-1G85R*^*M*^ animals. Inclusions in the middle image and right images were defined as small and medium size inclusions, respectively. Arrows point to inclusions. For oxidative stress trials, animals were treated with 2.5 mM paraquat for 3 hours. hSOD1WT-YFP was expressed under the pan-neuronal *snb-1* promoter. Scale Bar = 10 um. **(B)** Single-copy ALS *sod-1* mutations generated by MosSCI increased hSOD1WT-YFP inclusions compared to *sod-1WT*^*M*^ controls. Three independent trials. All inclusions were small in size. N > 30 for every genotype in every panel. Chi-square test: * *P* < 0.05; ** *P* < 0.01; *** *P* < 0.001 in all panels. Unless otherwise indicated, comparisons were made to *sod-1WT*^*M*^ animals. **(C)** *sod-1G85R*^*C*^ and *sod-1G93A*^*C*^ knock-in ALS model animals generated by CRISPR/Cas9 had increased hSOD1WT-YFP inclusions compared to *sod-1WT*^*C*^ wild type controls. Neither *sod-1L84V*^*C*^ nor loss of *sod-1* altered inclusions compared to *sod-1WT*^*C*^ and *sod-1(+)* controls, respectively. *sod-1(+)* designates the unedited wild type gene at the endogenous locus; this is the same allele present in standard N2 strain. All inclusions were small in size.**(D)** Paraquat-induced oxidative stress increased inclusions in *sod-1H71Y*^*M*^ and *sod-1G85R*^*M*^ model animals compared to *sod-1WT*^*M*^ wild type controls. Inclusions were scored manually as small, medium or large. If their diameters were more than four times larger than the small inclusions shown in Panel 2A, they were counted as large. **(E)** Paraquat-induced oxidative stress increased the percentage of animals with medium-size inclusions in *sod-1A4V*^*M*^, *sod-1H71Y*^*M*^ and *sod-1G85R*^*M*^ animals. Likewise, *empty*^*M*^ control animals had larger inclusions compared to *sod-1WT*^*M*^ controls. Inclusions were scored manually as small, medium or large, as described above.

ALS SOD1 mutations lead to formation of SOD1-rich proteinaceous inclusions in motor neurons [2–4]. Consistent with this observation, overexpression of human ALS SOD1 in *C*. *elegans* leads to formation of SOD1 inclusions in *C*. *elegans* neurons and muscles [13,23]. We found that expression of human wild type SOD1 tagged with YFP (hSOD1WT-YFP) resulted in small cytosolic inclusions in the ventral nerve cord motor neurons of a small fraction of wild type control animals (Fig 2A–2C). Next, we examined the impact of single-copy ALS *sod-1* models on neuronal inclusions formed by human SOD1WT-YFP in the same motor neurons. With the exception of *sod-1L84V*^*C*^, single-copy ALS SOD1 animals were more likely to have hSOD1WT-YFP inclusions compared to wild type control animals (Fig 2A–2C; *P* < 0.05, chi-square test). While single-copy ALS *sod-1* models increased the formation of hSOD1WT-YFP protein inclusions, loss of *sod-1* did not alter formation of hSOD1WT-YFP inclusions in these neurons (Fig 2B and 2C; *P* = 0.61 for *empty*^*M*^ vs *sod-1WT*^*M*^ and *P* = 0.12 for *sod-1(-)* vs *sod-1(+)*; *sod-1(+)* refers to the standard N2 wild type unedited allele). We conclude that loss of *sod-1* function does not alter propensity to form neuronal SOD1 inclusions in *C*. *elegans* motor neurons, but that most *C*. *elegans* single-copy ALS *sod-1* models show increased hSOD1WT-YFP inclusion formation.

Oxidative damage may induce SOD1 misfolding, resulting in aberrant protein accumulation [23,26]. Consequently, loss of *sod-1* function, coupled with an increase in oxidative damage, could exacerbate defects in ALS SOD1 models. To determine the impact of oxidative stress in single-copy ALS *sod-1* models, we exposed animals to paraquat, an oxidative stress inducing herbicide [27]. Loss of *sod-1* function decreased survival under paraquat-induced oxidative stress (S2A and S2B Fig, *P* < 0.001 for *empty*^*M*^ vs *sod-1WT*^*M*^ and *sod-1(-)* vs *sod-1(+)*, log-rank test), replicating a previously published finding [28]. Similarly, ALS knock-in alleles dramatically decreased survival under paraquat-induced oxidative stress, with the exception of *sod-1A4V*^*M*^ (S2A and S2B Fig; *P* < 0.01, log-rank test). By contrast, without external stress, survival was unaffected or slightly decreased in these genotypes (S1A and S1B Fig). We conclude that paraquat-induced oxidative stress may exacerbate or reveal survival defects in single-copy/knock-in models.

We also examined the impact of loss of *sod-1* function on formation of SOD1 inclusions after exposure to oxidative stress. Formation of hSOD1-YFP inclusions in ALS *sod-1* model animals and in animals lacking *sod-1* was measured after a brief period of paraquat exposure (3 hours). A significantly larger fraction of *sod-1H71Y*^*M*^ and *sod-1G85R*^*M*^ animals had more neuronal inclusions per animal compared to wild type controls (Fig 2D, *P* < 0.05, chi-square test). Moreover, *sod-1A4V*^*M*^, *sod-1H71Y*^*M*^ and *sod-1G85R*^*M*^ animals had larger neuronal inclusions compared to wild type controls (Fig 2E). Loss of *sod-1* function in *empty*^*M*^ controls lead to formation of bigger, but not more, neuronal inclusions (Fig 2D and 2E). In summary, ALS *sod-1* models lead to increased or accelerated formation of hSOD1WT-YFP protein inclusions, with the exception of *sod-1L84V*^*C*^. Loss of *sod-1* function did not initiate formation of hSOD1WT-YFP inclusions in *C*. *elegans* neurons, suggesting *sod-1* gain of function likely drives formation of hSOD1WT-YFP inclusions in ALS models.

Because hSOD1WT-YFP inclusions were not increased in the *C*. *elegans sod-1L84V*^*C*^ model, the utility of this model remains unclear. To be comprehensive in our analysis, we include *sod-1L84V*^*C*^ in all studies presented below.

### Oxidative stress leads to cholinergic motor neuron loss in *sod-1* ALS model animals, but not in animals lacking *sod-1* function

**Fig 3:**
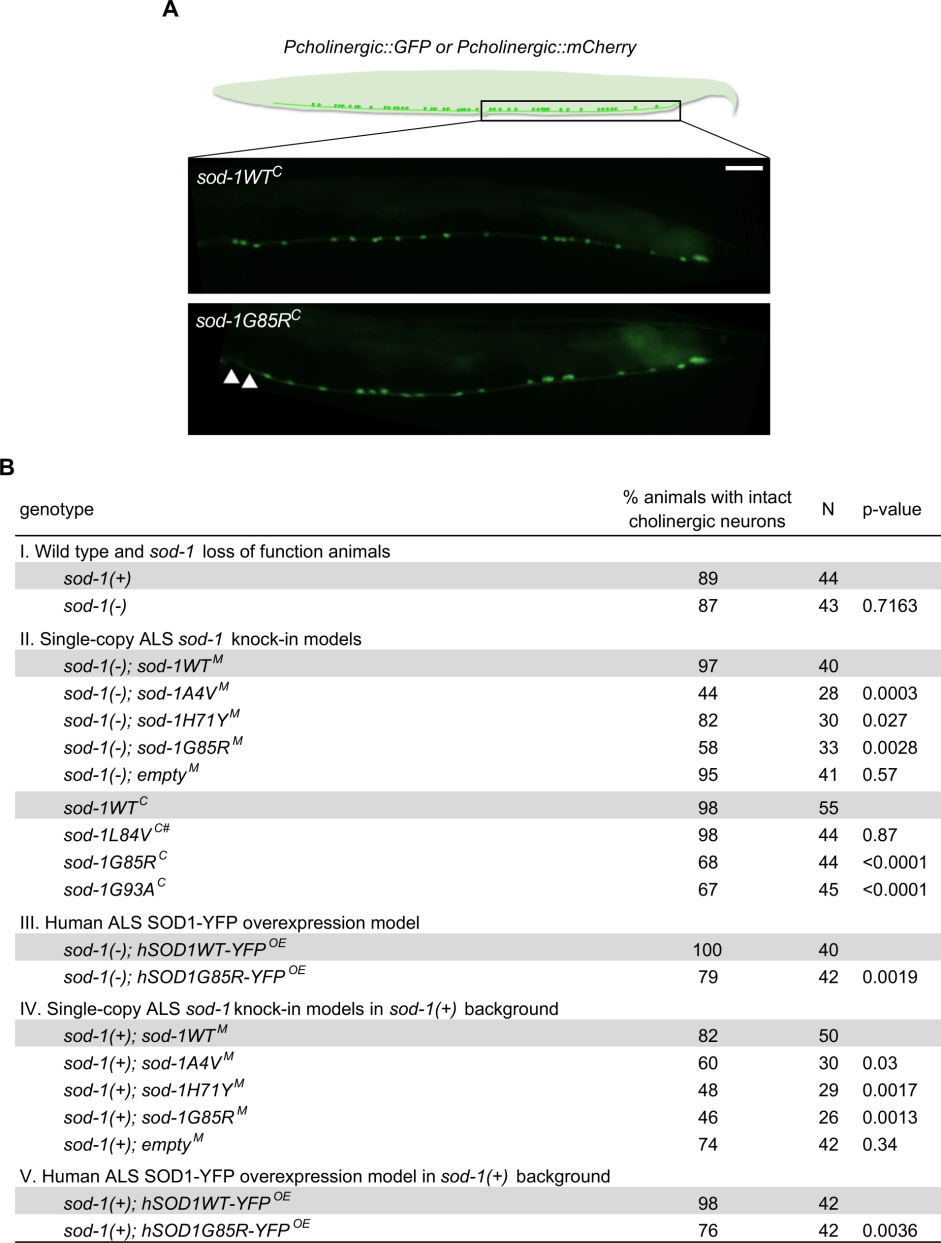
Gain of toxic function in *sod-1* drives oxidative stress induced cholinergic motor neuron degeneration in single-copy/knock-in ALS *sod-1* animals. **(A)** Cartoon illustration (top) and representative images (bottom) of cholinergic motor neurons posterior to vulva scored in neurodegeneration assays after overnight exposure to 2.5 mM paraquat-induced oxidative stress. Cholinergic motor neurons were visualized with *[unc-17p*::*GFP]*, except for human SOD1-YFP overexpression strains, which express YFP and were therefore scored with *[cho-1p*::*mCherry]*. Animals missing at least two neurons were scored as defective. White arrows point to missing neurons. N = number of animals tested. Chi-square test: * *P* < 0.05, ** *P* < 0.01, ** *P* < 0.001. **(B)** Animals were compared to appropriate wild type controls shown in the first row of each section. Cholinergic motor neurons were lost in *sod-1H71Y*^*M*^, *sod-1G85R*^*M*^, *sod-1G93A*^*C*^, *sod-1G85R*^*C*^ and *hSOD1G85R-YFP*^*OE*^ animals, but not in animals lacking *sod-1* function (*empty*^*M*^ controls and *sod-1(-)*) or in *sod-1L84V*^*C*^ animals (indicated with #). Presence of the endogenous wild type *sod-1(+)* allele in single-copy/knock-in models or in *hSOD1G85R-YFP*^*OE*^ overexpression models did not improve cholinergic motor neuron survival. *sod-1(+)* designates the unedited wild type gene at the endogenous locus; this is the same allele present in standard N2 strain.

Distinct neuronal populations are at greater risk for degeneration in ALS patients. Cholinergic and glutamatergic motor neurons are often disproportionately affected [29]. We examined the impact of ALS-associated mutations on cholinergic motor neuron survival in single-copy and overexpression models for ALS SOD1 in *C*. *elegans*. Survival of cholinergic motor neurons was assessed based on retention/loss of GFP or mCherry in animals carrying the *unc-17p*::*GFP* or *cho-1p*::*mCherry* transgenes, respectively, which express fluorescent proteins specifically in cholinergic neurons (Fig 3A). In the absence of oxidative stress, cholinergic motor neurons were intact in all examined strains (more than 30 animals per genotype scored with no neurons missing; [13]). However, overnight exposure to paraquat-induced cholinergic motor neuron degeneration in *sod-1A4V*^*M*^, *sod-1H71Y*^*M*^, *sod-1G85R*^*M*^, *sod-1G85R*^*C*^ and *sod-1G93A*^*C*^ animals, as well as in animals overexpressing the human SOD1G85R-YFP protein (*hSOD1G85R-YFP*^*OE*^), compared to appropriate wild type controls (Fig 3B, Part II and III; *P* < 0.05, chi-square test). Conversely, degeneration was not observed in control animals lacking *sod-1* or in *sod-1L84V*^*C*^ animals after paraquat treatment (Fig 3B, Part I and II). Between 2 to 4 motor neurons were lost in affected animals out of the 20 neurons scored in the posterior ventral nerve cord. These results suggest that loss of *sod-1* function is not sufficient to induce cholinergic motor neuron loss after exposure to oxidative stress. By contrast, most ALS mutations sensitize animals to oxidative stress and lead to cholinergic motor neuron loss.

The majority of ALS SOD1 patients carry a wild type SOD1 allele in addition to the mutated SOD1 allele. Consequently, we tested the impact of wild type endogenous *sod-1* on oxidative stress induced motor neuron degeneration. Crossing the endogenous unedited wild type *sod-1(+)* allele on chromosome II into *sod-1A4V*^*M*^, *sod-1H71Y*^*M*^ or *sod-1G85R*^*M*^ animals did not rescue paraquat-induced cholinergic motor neuron degeneration (Fig 3B, Part IV), consistent with a gain of toxic function mechanism in ALS *sod-1* alleles. Similarly, crossing *sod-1(+)* into the *hSOD1G85R-YFP*^*OE*^ animals failed to rescue stress induced degeneration (Fig 3B, Part V). Thus, in both single-copy and overexpression ALS SOD1 models, SOD1 gain of toxic function and oxidative stress results in cholinergic neurodegeneration.

### NMJ functional defects differ in single-copy *versus* overexpression models

**Fig 4:**
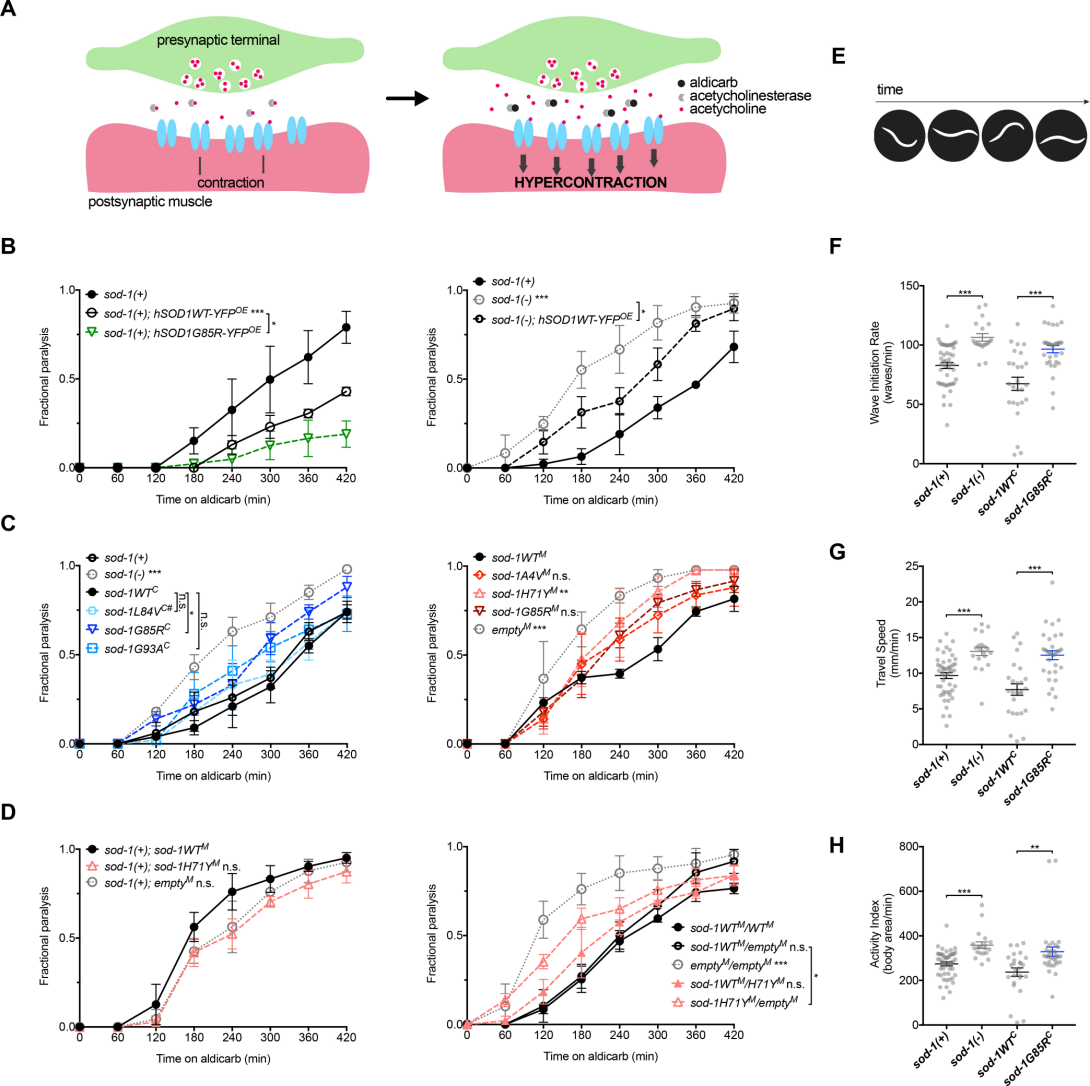
Neuromuscular junction activity differs in overexpression *versus* single-copy/knock-in models. **(A)** The drug aldicarb inhibits acetylcholinesterase and leads to acetylcholine build-up at the neuromuscular junction (NMJ) with a characteristic time course for paralysis in young adult animals. Either resistance or hypersensitivity to aldicarb indicates defective neuromuscular signaling. Increased NMJ activity can accelerate paralysis as acetylcholine accumulates more rapidly, while decreased NMJ activity can slow paralysis. **(B)** Left panel: Neuronal overexpression of human SOD1WT-YFP protein (*hSOD1WT-YFP*^*OE*^) results in aldicarb resistance, compared to non-transgenic wild type *sod-1(+)* control animals (standard N2 strain). Animals overexpressing human SOD1G85R-YFP (*hSOD1G85R-YFP*^*OE*^) were even more resistant to aldicarb than animals overexpressing human SOD1WT-YFP protein (*hSOD1WT-YFP*^*OE*^). Right panel: *sod-1(-)* animals were hypersensitive to aldicarb compared to wild type *sod-1(+)* controls. Neuronal overexpression of wild type human SOD1-YFP partially rescues aldicarb response in *sod-1(-)* animals. Fractional paralysis refers to the fraction of animals paralyzed at a given time point. Three independent trials in B-D. N > 30 for each genotype in all panels. Error bars indicate ±SEM. Log-rank test: * *P* < 0.05; ** *P* < 0.01; *** *P* < 0.001 in all panels. **(C)** Single-copy ALS *sod-1* knock-in models and controls generated by MosSCI were tested in the *sod-1(-)* background for aldicarb resistance. *sod-1H71Y*^*M*^ animals, like *sod-1* null *empty*^*M*^ controls, were hypersensitive to aldicarb compared to *sod-1WT*^*M*^ wild type controls. While *sod-1G85R*^*M*^ and *sod-1A4V*^*M*^ models appear slightly hypersensitive, this difference was not significant. Single-copy ALS *sod-1* knock-in models and their appropriate wild type controls generated by CRISPR/Cas9 were tested for aldicarb resistance. *sod-1L84V*^*C*^ indicated with #. *sod-1G85R*^*C*^ animals and *sod-1(-)* animals were hypersensitive to aldicarb, compared to *sod-1WT*^*C*^ and *sod-1(+)* wild type controls (standard N2 strain), respectively. **(D)** Presence of the endogenous wild type *sod-1(+)* allele in *sod-1H71Y*^*M*^ animals and *empty*^*M*^ controls rescues aldicarb response, compared to *sod-1WT*^*M*^ animals in the same background. Heterozygous *sod-1WT*^*M*^*/H71Y*^*M*^ animals had normal aldicarb response compared to homozygous *sod-1WT*^*M*^*/WT*^*M*^ wild type controls. **(E-H)** Assessment of swimming in young adults animals after paraquat treatment was examined with computer vision software CeLeST [31]. Panel E illustrates body bends while swimming. *sod-1G85R*^*C*^ and *sod-1(-)* animals had increased Wave Initiation Rate (Panel F), Travel Speed (Panel G) and Activity Index (Panel H) compared to their *sod-1WT*^*C*^ and *sod-1(+)* controls, respectively. *sod-1(+)* wild type controls are the standard N2 strain. Two independent trials. N > 20 for each genotype. Error bars indicate ±SEM. Kruskal-Wallis test: * *P* < 0.05; ** *P* < 0.01; *** *P* < 0.001.

Neuromuscular junction (NMJ) dysfunction is an early defect in ALS patients [30]. Heterologous overexpression of human SOD1G85R in *C*. *elegans* neurons also leads to NMJ dysfunction [13], based on resistance to aldicarb, an inhibitor of acetylcholinesterase. Exposure to aldicarb leads to acetylcholine buildup at the NMJ, with the consequent hyperexcitation of postsynaptic muscles and paralysis over a characteristic time course (Fig 4A). Either hypersensitivity or resistance to aldicarb indicates defective neuromuscular signaling and suggests increased or decreased overall NMJ cholinergic signaling.

We confirmed that neuronal overexpression of the human mutant SOD1G85R causes aldicarb resistance, compared to animals overexpressing the human wild type SOD1 protein (Fig 4B, left panel; *P* < 0.05, log-rank test and [13]). However, we found that both transgenic strains were more resistant to paralysis by aldicarb than non-transgenic wild type *C*. *elegans* (Fig 4B, left panel). Thus, overexpression of wild type human SOD1 is deleterious and causes defects in *C*. *elegans* NMJ function.

Next, we examined the impact of single-copy ALS models on NMJ function. The response of wild type control animals was indistinguishable from non-transgenic *C*. *elegans* (Fig 4C). And, in single-copy *sod-1A4V*^*M*^, *sod-1L84V*^*C*^, *sod-1G85R*^*M*^ and *sod-1G93A*^*C*^ animals, response to aldicarb was normal (Fig 4C; *sod-1A4V*^*M*^ vs *sod-1WT*^*M*^, *P* = 0.20; *sod-1L84V*^*C*^ vs *sod-1WT*^*C*^, *P* = 0.76; *sod-1G85R*^*M*^ vs *sod-1WT*^*M*^, *P* = 0.071; *sod-1G93A*^*C*^ vs *sod-1WT*^*C*^, *P* = 0.44, log-rank test). However, *sod-1G85R*^*C*^ and *sod-1H71Y*^*M*^ animals were hypersensitive to aldicarb compared to wild type controls (Fig 4C; *P* < 0.05, log-rank test). Thus, even though single-copy models are not uniform in their response to aldicarb, none of these strains were resistant to aldicarb-induced paralysis. Overall, we observed dramatically different consequences for *C*. *elegans* NMJ function in overexpression *versus* single-copy ALS model animals.

The impact of *sod-1* loss of function on *C*. *elegans* NMJ function has not been reported previously. We found that loss of endogenous *sod-1* function lead to aldicarb hypersensitivity, compared to standard N2 strain *sod-1(+)* controls (Fig 4C, left panel; *P* < 0.001 for *sod-1(-)* vs *sod-1(+)*). To confirm this change was due to decreased *sod-1* activity, and to establish conservation of SOD1 function across species, we undertook phenotypic rescue studies. Introduction of *C*. *elegans sod-1WT*^*M*^ to *sod-1(-)* fully restored normal response to aldicarb (Fig 4C, right panel; *sod-1WT*^*M*^ vs *empty*^*M*^), and introduction of neuronal human SOD1WT-YFP partially restored aldicarb response (Fig 4B, right panel; *sod-1(-); hSOD1WT-YFP*^*OE*^ vs *sod-1(-)*). These results suggest that NMJ functional defects in ALS *sod-1* model animals may be, in part, driven by loss of *sod-1* function.

To confirm that *sod-1* loss of function contributes to NMJ defects, we also examined the consequences of altering *sod-1* dosage. First, we confirmed that restoring *sod-1* function would restore normal NMJ function in *sod-1H71Y*^*M*^ animals. We crossed the unedited endogenous *sod-1(+)* allele into *sod-1H71Y*^*M*^ allele background. These animals had normal response to aldicarb (Fig 4D, left panel; *sod-1(+); sod-1H71Y*^*M*^ vs *sod-1(+); sod-1WT*^*M*^, *P* = 0.05).

To confirm that aldicarb hypersensitivity in ALS *sod-1* animals is driven primarily by *sod-1* loss of function, we examined aldicarb response in animals heterozygous for ALS *sod-1* transgenes. Homozygous ALS *sod-1* animals were crossed to homozygous *sod-1WT*^*M*^ or *empty*^*M*^ males carrying a GFP-expressing transgene. Then, the heterozygous GFP-positive cross-progeny were examined for aldicarb resistance after paraquat treatment. As control, we confirmed that *empty*^*M*^/*empty*^*M*^ cross-progeny were hypersensitive to aldicarb compared to *sod-1WT*^*M*^*/sod-1WT*^*M*^ cross-progeny (Fig 4D, right panel).

Introduction of one functional copy of *sod-1* yielded *sod-1WT*^*M*^/*empty*^*M*^ animals, which were normal in response and indistinguishable from *sod-1WT*^*M*^*/WT*^*M*^ animals (Fig 4D, right panel; *sod-1WT*^*M*^*/WT*^*M*^ vs *sod-1WT*^*M*^*/empty*^*M*^, *P* = 0.16). Next, we determined if the *sod-1H71Y*^*M*^ aldicarb response defect is recessive. We found that heterozygous *sod-1WT*^*M*^*/H71Y*^*M*^ animals had normal aldicarb response (Fig 4D, *sod-1WT*^*M*^*/H71Y*^*M*^ vs *sod-1WT*^*M*^*/WT*^*M*^, *P* = 0.23). Taken together, these findings suggest that loss of *sod-1* function contributes to the NMJ functional defects in both *sod-1(-)* and *sod-1H71Y*^*M*^ animals.

NMJ functional defects are often associated with defective localization of NMJ presynaptic proteins. A previous study has reported defective presynaptic synaptobrevin/SNB-1 localization at the NMJ of the human SOD1G85R overexpression animals [13]. To assess the impact of ALS *sod-1* models on NMJ proteins, we determined the localization and intensity of fluorescently-labelled pre-synaptic proteins in live animals using previously described transgenic strains and protocols in adult *sod-1H71Y*^*M*^ and *sod-1G85R*^*M*^ animals. Size, distribution and intensity of presynaptic synaptobrevin/SNB-1 and intersectin-1/ITSN-1 fluorescent punctae were normal in *sod-1H71Y*^*M*^ and *sod-1G85R*^*M*^ animals (S3A and S3B Fig). Unlike overexpression model animals, presynaptic protein levels and localization were not different in single-copy ALS *sod-1* animals. While this approach may not reveal subtle defects in NMJ structure, we conclude that *sod-1H71Y*^*M*^ and *sod-1G85R*^*M*^ have no major impact on the NMJ structure or number in *C*. *elegans*.

Considering that oxidative stress exacerbated defects in single-copy ALS *sod-1* animals, we examined the impact of this stress on locomotion. Adult *sod-1G85R*^*C*^ animals were treated overnight with paraquat and tested for swimming locomotion (Fig 4E) using computer vision software CeleST [31]. Increased locomotion activity was observed in *sod-1G85R*^*C*^ and *sod-1(-)* animals compared to appropriate wild type controls (Fig 4F–4H, *P* < 0.05, Kruskal-Wallis test); Activity Index, Travel Speed, and Wave Initiation Rate metrics were all increased, consistent with increased activity. Combined, NMJ defects and locomotion assays results suggest that loss of *sod-1* function may increase NMJ signaling, without dramatic perturbation in synapse structure and number. Cholinergic motor neuron defects might be expected to change locomotion in *sod-1G85R*^*M*^ model animals; we found that oxidative stress also increased their locomotion activity, compared to controls. At first glance, this is counterintuitive as cholinergic motor neuron degeneration should impair locomotion. However, modest loss of cholinergic motor neurons does not dramatically impair *C*. *elegans* locomotion based on laser ablation studies [32,33]. Additionally, paraquat is a strongly aversive stimulus and *C*. *elegans* can respond to noxious environments with a coordinated escape response by decreasing spontaneous reversals and increasing forward locomotion speed [34]. Consistent with this, we observed that placing *sod-1G85R*^*M*^, *sod-1H71Y*^*M*^ *sod-1G85R*^*C*^, or *sod-1* loss of function animals overnight on culture dishes containing paraquat resulted in high rates of escape; after 24 hours, roughly 40% of *sod-1H71Y*^*M*^, 47% of *empty*^*M*^, and 38% of *sod-1G85R*^*M*^ animals left the 2.5mM paraquat culture dishes, compared to only 2% of *sod-1WT*^*M*^ control animals. Similarly, 23% of *sod-1G85R*^*C*^ animals left after overnight exposure to paraquat, compared to only 1% of *sod-1WT*^*C*^ animals (3 trials, >80 animals per genotype total). Combined, our results suggest that the increased locomotion activity observed in *sod-1G85R*^*C*^ animals is likely an escape response and that single-copy ALS *sod-1* models differ significantly from previously described human SOD1 overexpression models in their impact on NMJ function.

### Dopaminergic, serotonergic and GABAergic neurons were relatively spared in single-copy ALS model animals, but glutamatergic neurons were lost

Neuron loss in ALS patients is generally limited to specific subsets of cholinergic and glutamatergic neurons [29]. We examined the specificity of neurodegeneration in single-copy/knock-in ALS model animals. Neurotransmitter-type specific GFP reporter constructs for glutamatergic, dopaminergic, serotonergic, and GABAergic neurons were used to facilitate scoring of neuron loss, *i*.*e*. *dat-1p*::*GFP*, *tph-1*::*GFP*, *unc-47p*::*GFP*, and *osm-11p*::*GFP*. As the single-copy *sod-1G85R* models show robust cholinergic motor neuron loss after paraquat stress, this genetic model was used to assess loss of other GFP-labelled neurons after paraquat-induced oxidative stress. For dopaminergic neurons, all four CEPs and both ADE neurons were scored, as well as ADE neuron sensory processes. For serotonergic neurons, both NSM and both ADF neurons were scored. For GABAergic neurons, nineteen GABAergic motor neurons in the ventral cord were scored. For glutamatergic neurons, both ASH neurons were scored.

**Fig 5:**
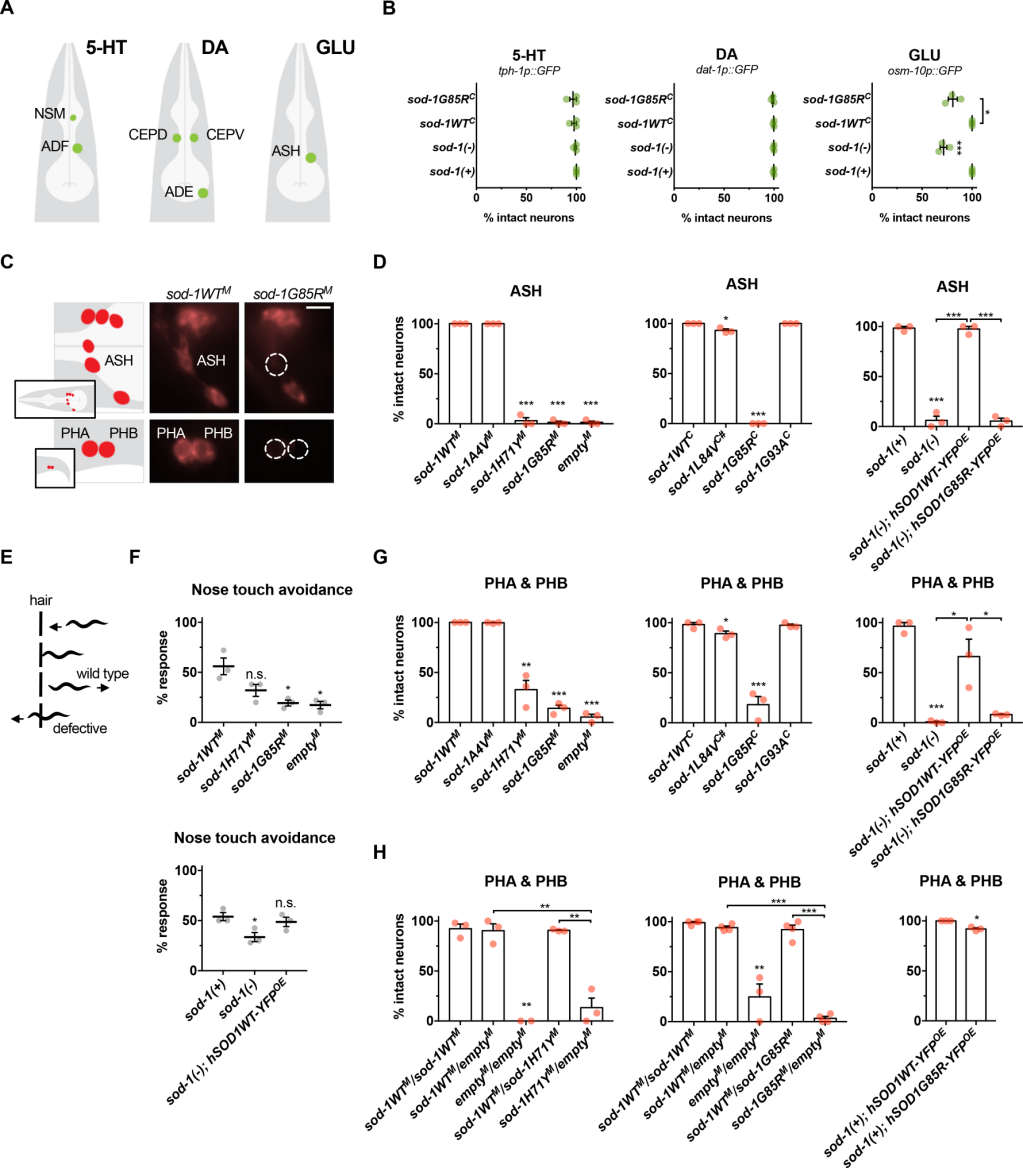
*sod-1* loss of function drives oxidative stress induced glutamatergic neuron degeneration in single-copy/knock-in ALS *sod-1* animals. **(A)** Illustration of glutamatergic (*osm-10p*::*GFP*), serotonergic (*tph-1p*::*GFP*) and dopaminergic (*dat-1p*::*GFP*) neurons examined to assess neurodegeneration after paraquat-induced oxidative stress. **(B)** Serotonergic (5-HT) and dopaminergic (DA) neurons were unaffected in all genotypes examined. However, glutamatergic neurons (GLU) were lost after paraquat-induced oxidative stress in *sod-1(-)* and *sod-1G85R*^*C*^ animals, compared to *sod-1(+)* and *sod-1WT*^*C*^ controls, respectively. *sod-1(+)* designates the unedited wild type gene at the endogenous locus; this is the same allele present in standard N2 strain. **(C)** Left: illustration of six glutamatergic sensory head neurons (top) and two tail neurons (bottom) labelled with lipophilic retrograde dye DiD. Images show right side of animal; similar neurons on left side were also scored. Middle: representative images of *sod-1WT*^*M*^ animals with intact glutamatergic sensory neurons in the head (top) and tail (bottom) labelled with DiD after paraquat treatment. Right: representative images of *sod-1G85R*^*M*^ animals; ASH (top), PHA and PHB (bottom) neurons fail to take up dye only after paraquat treatment, indicating either process degeneration/retraction or neuronal loss. Scale bar represents 10 μm. **(D)** Percent intact ASH neurons in ALS model animals after paraquat treatment, compared to appropriate *sod-1WT*^*M*^, *sod-1WT*^*C*^, *hSOD1WT-YFP*^*OE*^ *or sod-1(+)* wild type controls. Degeneration in ASH neurons was increased in *sod-1H71Y*^*M*^, *sod-1G85R*^*M*^, *sod-1L84V*^*C*^, *sod-1G85R*^*C*^ and *hSOD1G85R-YFP*^*OE*^ animals, as well as in animals lacking *sod-1* function (*empty*^*M*^ controls and *sod-1(-)*). *sod-1L84V*^*C*^ indicated with #. *sod-1(+)* is the standard N2 strain. In all figure panels, results of three independent trials are shown. Error bars indicate ±SEM. N > 25 for all genotypes in all panels. Two-tailed t-test: * *P* < 0.05; ** *P* < 0.01; *** *P* < 0.001. **(E)** Illustration of the nose-touch avoidance assay. Wild type animals initiate backward locomotion after their nose contacts a hair; animals that cross over the hair or fail to reverse are scored as defective. ASH sensory neurons play an important role in this behavior [39]. Animals were treated with 2.5 mM paraquat for 4 hours before the nose touch response assay. After 4 hours of paraquat treatment, nose touch response was modestly impaired in wild type controls (57% response), even though 98% of ASH neurons were intact (3 trials, 35 animals total). After the same 4 hour treatment, nose touch response was severely impaired in *sod-1G85R*^*M*^ animals (28% response), while 49% of ASH neurons were intact (3 trials, 35 animals total). **(F)** Top: *sod-1G85R*^*M*^ animals and *empty*^*M*^ control animals lacking *sod-1* function were defective in response to nose-touch after paraquat treatment compared to *sod-1WT*^*M*^ control animals. Bottom: Neuronal expression of the human SOD1WT-YFP in *sod-1(-)* animals restored nose-touch response. *sod-1(+)* is standard N2 strain. **(G)** Percent intact glutamatergic PHA and PHB neurons in ALS model animals after paraquat treatment, compared to appropriate *sod-1WT*^*M*^, *sod-1WT*^*C*^, *or sod-1(+)* wild type control animals. Degeneration was increased in PHA and PHB neurons of *sod-1H71Y*^*M*^, *sod-1G85R*^*M*^ and *sod-1G85R*^*C*^ animals compared to appropriate wild type controls. Loss of *sod-1* function in *empty*^*M*^ control and *sod-1(-)* animals also led to PHA and PHB degeneration, compared to *sod-1WT*^*M*^ and *sod-1(+)*. *sod-1(+)* is the standard N2 strain. Neuronal overexpression of human SOD1WT-YFP, but not human SOD1G85R-YFP, partially restored dye-uptake in *sod-1(-)* animals. **(H)** To examine the consequences of altering gene dosage and assess recessive/dominance of single-copy/knock-in ALS alleles, homozygous ALS *sod-1* animals and controls were crossed to homozygous *sod-1WT*^*M*^ or *empty*^*M*^ males carrying a GFP-expressing transgene, and cross-progeny were tested for DiD dye-uptake after paraquat treatment. *empty*^*M*^/*empty*^*M*^ cross-progeny had defects after paraquat treatment, compared to *sod-1WT*^*M*^*/WT*^*M*^ cross-progeny, while *sod-1WT*^*M*^*/empty*^*M*^ animals had intact glutamatergic neurons. Heterozygous *sod-1H71Y*^*M*^*/empty*^*M*^ and *sod-1G85R*^*M*^*/empty*^*M*^ animals were defective, compared to heterozygous *sod-1WT*^*M*^*/empty*^*M*^ animals. By contrast, *sod-1WT*^*M*^*/sod-1H71Y*^*M*^ or *sod-1WT*^*M*^*/sod-1G85R*^*M*^ animals had intact glutamatergic neurons after paraquat treatment. Additionally, animals overexpressing the human hSOD1G85R-YFP protein in the *sod-1(+)* background had low penetrance dye-filling defects. *sod-1(+)* designates the unedited wild type gene at the endogenous locus; this is the same allele present in standard N2 strain.

A previous study in *C*. *elegans* found significant dopaminergic neuron degeneration in younger animals after a more extended exposure period to paraquat [35]. However, we found no dopaminergic or serotonergic neuron loss in *sod-1G85R*^*C*^, *sod-1(-)*, or control animals after paraquat-induced oxidative stress (Figs 5A, 5B, S4A and S4B), and dopaminergic ADE neuron sensory processes did not degenerate after paraquat treatment (S4B Fig). We conclude that neither *sod-1G85R*^*C*^ nor *sod-1* loss leads to loss of dopaminergic or serotonergic neurons in single-copy ALS animals.

*C*. *elegans* have both cholinergic and GABAergic motor neurons that directly synapse onto muscles; these neurons coordinately and reciprocally regulate muscle excitability [36]. After paraquat treatment, roughly 25% of *sod-1G85R*^*M*^ animals had GABAergic motor neuron loss, while *sod-1WT*^*M*^ or *sod-1(-)* animals had less than 10% affected animals (S4D Fig). No GABAergic motor neurons were lost in more than 30 *sod-1G85R*^*M*^ animals tested without paraquat stress. Extending this analysis, we examined GABAergic motor neuron survival in *sod-1A4V*^*M*^ and *sod-1H71Y*^*M*^ animals. Less than 20% and 10% of animals lost GABAergic motor neurons after paraquat treatment in these genotypes, respectively, which was not significant (S4D Fig). We conclude that GABAergic motor neurons are affected only in the single-copy *sod-1G85R*^*M*^ model.

A subset of ALS patients have glutamatergic cortical motor neuron degeneration and/or sensory neuropathy [5–7,29]. After paraquat treatment, approximately 20% of the glutamatergic ASH sensory neurons were lost in *sod-1G85R*^*C*^ animals (S4C Fig). Similarly, about 30% of the glutamatergic ASH sensory were lost in *sod-1(-)* animals after paraquat treatment, while virtually all neurons were intact in *sod-1WT*^*C*^ animals (S4C Fig). No neurons were lost without paraquat stress in any genotype. These results suggested that glutamatergic neurons are also lost in the single-copy *sod-1G85R*^*C*^ ALS model animals. Combined with the results above, we find that neuron loss occurs in *sod-1G85R*^*C*^ animals after oxidative stress, and primarily affects motor neurons and glutamatergic neurons, with relative sparing of serotonergic and dopaminergic neurons.

### Loss of *sod-1* function leads to glutamatergic neuron degeneration after oxidative stress

Degeneration of neuronal processes has been previously reported in a single-copy ALS SOD1 mouse model and in other *C*. *elegans* models of neurodegenerative disease [20,37]. In the head and tail, *C*. *elegans* have glutamatergic sensory neuron processes that are exposed to the environment, which allows uptake of lipophilic fluorescent dyes, such as DiD (C_67_H_103_ClN_2_O_3_S). Degenerative sensory process loss or cell death completely stops dye uptake by individual neurons, as shown in *C*. *elegans* models of Huntington’s Disease polyglutamine toxicity [37]. Without paraquat-induced oxidative stress, dye uptake was normal in all *C*. *elegans* ALS *sod-1* model animals and in animals lacking *sod-1* function (more than 30 animals scored per genotype, more than two trials). ASH, PHA and PHB neurons failed to take up DiD in *sod-1(-)* animals after paraquat-induced oxidative stress (Fig 5D and 5G), but DiD uptake was normal in five other classes of glutamatergic neurons. Single-copy/knock-in *sod-1H71Y*^*M*^, *sod-1G85R*^*M*^, *sod-1G85R*^*C*^ and *sod-1L84V*^*C*^ animals had defective dye uptake in the same neurons after exposure to oxidative stress, although the penetrance of this defect varied between genotypes with little degeneration in *sod-1L84V*^*C*^ (Fig 5C, 5D and 5G). Again, five other classes of glutamatergic neurons that take up DiD were unaffected in all of these genotypes. No dye uptake defects were observed in *sod-1A4V*^*M*^ and *sod-1G93A*^*C*^ animals, even after paraquat treatment (Fig 5D and 5G).

The results above suggest that decreased *sod-1* function renders glutamatergic neurons hypersensitive to oxidative stress. To test this hypothesis, we undertook phenotypic rescue experiments. As SOD-1 function is conserved across species, we crossed the previously described transgene expressing human wild type SOD1-YFP in *C*. *elegans* neurons into *sod-1(-)* animals. DiD uptake defects were dramatically reduced in the resulting *sod-1(-); hSOD1WT-YFP*^*OE*^ animals (Fig 5G, right), consistent with conserved dismutase activity and loss of *sod-1* function contributing to this defect. The G85R missense allele is reported to dramatically decrease human SOD1 dismutase activity [38]. We found that crossing the *hSOD1G85R-YFP*^*OE*^ transgene into *sod-1(-)* animals did not ameliorate the dye uptake defects (Fig 5G, right). We conclude that decreased *sod-1* function can lead to oxidative stress induced neurodegeneration of glutamatergic neurons in *C*. *elegans*.

To confirm that glutamatergic neurodegeneration in ALS *sod-1* models is driven primarily by *sod-1* loss of function, we examined glutamatergic neurodegeneration in animals heterozygous for ALS *sod-1* transgenes. Homozygous ALS *sod-1* animals were crossed to homozygous *sod-1WT*^*M*^ or *empty*^*M*^ males carrying a GFP-expressing transgene. Then, the heterozygous GFP-positive cross-progeny were examined for glutamatergic neuron degeneration after paraquat treatment. As control, we confirmed that *empty*^*M*^/*empty*^*M*^ cross-progeny had dye-uptake defects, compared to *sod-1WT*^*M*^*/sod-1WT*^*M*^ cross-progeny. (PHA and PHB in Fig 5H, ASH in S4E Fig). Animals carrying a single *sod-1WT*^*M*^ allele (*sod-1WT*^*M*^*/empty*^*M*^) had no degeneration, confirming that loss of *sod-1* function is not dominant. When we examined animals hemizygous for *sod-1H71Y*^*M*^ or *sod-1G85R*^*M*^ (*sod-1H71Y*^*M*^*/empty*^*M*^ and *sod-1G85R*^*M*^*/empty*^*M*^), they had ASH, PHA and PHB dye-uptake defects after paraquat treatment, suggesting that these alleles cause *sod-1* loss of function (Figs 5H and S4E). Consistent with this, dye-uptake defects were not seen in *sod-1WT*^*M*^*/sod-1H71Y*^*M*^ or *sod-1WT*^*M*^*/sod-1G85R*^*M*^ animals, (*sod-1WT*^*M*^*/ALS allele* vs *empty*^*M*^*/ALS allele*, Figs 5H and S4E). Combined, these studies suggest that *H71Y* and *G85R* decrease *sod-1* function, leading to stress induced degeneration in a subset of glutamatergic neurons.

Both SOD1 loss and gain of function in ALS may contribute to degeneration and their relative contributions may shift in different neuronal subtypes. Overexpression models are ideally suited to examine gain of function consequence of disease alleles. We assessed the consequences of overexpressing wild type or mutant human SOD1 in the glutamatergic neurons of otherwise normal *C*. *elegans*. Degeneration was not observed in *hSOD1WT-YFP*^*OE*^ animals when the unedited endogenous *sod-1(+)* allele was present, based on dye-filling after paraquat treatment. By contrast, a modest level of degeneration was observed in *sod-1(+); hSOD1G85R-YFP*^*OE*^ animals (Fig 5H, right). This result is consistent with a deleterious impact of overexpressed human SOD1 G85R, which may antagonize endogenous *sod-1* or have a novel, toxic gain of function.

Neurodegenerative diseases lead to behavioral changes. *C*. *elegans* ASH neurons are critical for eliciting a mechanosensory response when the nose contacts a physical barrier during forward locomotion. Loss of both ASH neurons eliminates ~60% of this behavioral response, but response is intact if one ASH neuron is present [39]. We examined mechanosensory response after exposure to paraquat and found defective nose touch avoidance response in *sod-1G85R*^*M*^ and *empty*^*M*^ animals (Fig 5E and 5F). This defective response is consistent with dye uptake defects and neuronal loss observed in these genotypes. We also undertook a behavioral rescue experiment. Animals expressing neuronal hSOD1WT-YFP had normal nose touch avoidance response compared to non-transgenic control animals (Fig 5F, bottom). These results suggest that nose-touch avoidance defects in ALS *sod-1* model animals may be, in part, driven by loss of *sod-1* function.

## Discussion

Herein, we report the first single-copy/knock-in models for ALS SOD1 in *C*. *elegans* generated using targeted genome editing. A4V, H71Y, L84V, G85R and G93A patient amino acid changes were introduced into the *C*. *elegans sod-1* gene, using two different strategies. We found that *C*. *elegans* single-copy/knock-in models for A4V, H71Y, G85R and G93A accelerated accumulation of the YFP-tagged wild type human SOD1 protein in *C*. *elegans* motor neurons. We also found that SOD1 mutations differentially impact glutamatergic and cholinergic neurons in *C*. *elegans*. A4V, G85R, H71Y and G93A lead to oxidative stress induced loss of cholinergic motor neurons, while L84V, H71Y and G85R lead to oxidative stress induced degeneration of glutamatergic neurons. Other neuronal populations (dopaminergic and serotonergic) were relatively spared, suggesting that single-copy ALS SOD1 knock-in models in *C*. *elegans* may recapitulate the selective sensitivity of cholinergic and glutamatergic neurons in ALS caused by SOD1 mutations. Furthermore, we found that *sod-1* loss of function is a major contributor to glutamatergic neuron degeneration after oxidative stress. However, *sod-1* gain of function likely drives cholinergic motor neuron degeneration. Combined, these results suggest that *C*. *elegans* knock-in models, at a minimum, complement overexpression models and provide unique insights into why different SOD1 mutations lead to degeneration in different types of neurons.

Cytoplasmic SOD1 aggregates are found in ALS SOD1 patients and in most ALS SOD1 overexpression models [15,23,40–42]. *C*. *elegans* single-copy/knock-in models for A4V, H71Y, G85R and G93A increased accumulation of the YFP-tagged human wild type SOD1 protein in small cytosolic inclusions within *C*. *elegans* motor neurons, even under optimal growth conditions. These hSOD1WT-YFP inclusions were possibly seeded by misfolded mutant *C*. *elegans* SOD-1 protein in single-copy/knock-in models. Are these inclusions caused by loss or gain of *sod-1* function? Because inclusions were not increased in *C*. *elegans* with decreased *sod-1* function, this is likely a gain of function defect. It seems likely that introduction of the ALS patient amino acid changes into the *C*. *elegans* SOD-1 protein conferred a neomorphic/novel gain of function that increased inclusion propensity. In contrast with these four alleles, we found that hSOD1WT-YFP inclusions were not increased in the motor neurons of *sod-1L84V*^*C*^ animals. Previous studies have shown that driving human L84V SOD1 expression in cells or transgenic mice results in SOD1 aggregation [40,43]. The failure of *sod-1L84V*^*C*^ to increase inclusions in *C*. *elegans* neurons may arise from differences between human and *C*. *elegans* SOD1 proteins. Consequently, the usefulness of the *C*. *elegans sod-1L84V*^*C*^ model remains unclear.

ALS is characterized by degeneration of lower cholinergic motor neurons. However, a significant fraction of patients present with cortical glutamatergic neuron degeneration [44]. Given that cholinergic motor neurons always degenerate and the technical challenges of studying cortical neurons, most work in the ALS field focuses on cholinergic motor neurons. In overexpression ALS SOD1 mice and in other models, high level expression of ALS SOD1 patient alleles leads to degeneration that has been ascribed to a SOD1 neomorphic/novel toxic gain of function [15,41]. This is consistent with the observation that most ALS SOD1 alleles are dominant in patients. Under standard culture conditions, *C*. *elegans* cholinergic motor neurons do not die in young adult animals, in either single-copy/knock-in models described herein or in human SOD1 ALS models overexpression models described previously [13]. We found that cholinergic motor neurons in all single-copy/knock-in ALS *sod-1* models were hypersensitive to oxidative stress, except *sod-1L84V*^*C*^. Oxidative stress lead to motor neuron loss in A4V, H71Y, G85R and G93A single-copy/knock-in animals, and in *hSOD1G85R-YFP*^*OE*^ overexpression model animals [13], which has not been reported previously. Motor neurons were not lost in animals lacking *sod-1* function and introduction of wild type *sod-1* did not rescue cholinergic motor neuron degeneration in A4V, H71Y or G85R single-copy/knock-in models. Oxidative stress exposure also increased the number and/or size of neuronal SOD1 inclusions in these ALS SOD1 models. These results are consistent with previous work showing misregulation of cellular stress response pathways in ALS SOD1 models [45] and increased SOD1 aggregation after oxidative stress [23,46]. Combined, these results suggest that A4V, H71Y, G85R and G93A SOD1 ALS alleles confer a toxic gain of function that is deleterious to *C*. *elegans* cholinergic neurons.

Defects in glutamatergic corticospinal tracts can be detected in many ALS patients [5,29] and glutamatergic sensory neurons are also lost in patients [6,7]. In *C*. *elegans* single-copy/knock-in G85R and H71Y SOD1 ALS model animals, glutamatergic neurons were also hypersensitive to oxidative stress; paraquat treatment led to degeneration of neurons in these animals, but not in control animals. Similarly, loss of *sod-1* function resulted in glutamatergic neuron degeneration after oxidative stress. The defects in H71Y and G85R single-copy/knock-in animals are recessive and introduction of hSOD1WT-YFP ameliorated defects in *sod-1(-)* animals. Combined, these results suggest that oxidative stress hypersensitivity in glutamatergic neurons is a *C*. *elegans sod-1* loss of function defect. This is consistent with studies in mice; SOD1 loss of function results in degenerative changes [38,47]. The neurodegeneration observed here in *C*. *elegans* glutamatergic and cholinergic neurons stands in contrast to the lack of neurodegeneration observed in dopaminergic and serotonergic neurons under the same oxidative stress conditions. Additionally, even after oxidative stress, *C*. *elegans* GABAergic motor neurons are relatively spared in single-copy/knock-in models. This level of specificity is unusual and suggests that the mechanisms underlying ALS specificity for specific neuronal classes/neurotransmitter subtypes may be recapitulated in *C*. *elegans* knock-in models.

Work with overexpression models has been critical for the field and has definitively shown that ALS SOD1 proteins have toxic gain of function properties. However, use of overexpression models is subject to two caveats. First, overexpression models compare the toxic effects of the mutant SOD1 protein to the effects of overexpressing the wild type protein. We and others find that overexpressing wild type SOD1 has deleterious consequences [17,41,48]. Increased levels of wild type human SOD1 alters NMJ function in *C*. *elegans*; *hSOD1WT-YFP*^*OE*^ animals paralyze more slowly than normal animals in the presence of aldicarb (Fig 4B). And, increased levels of human wild type SOD1 protein in *C*. *elegans* neurons decreased survival under paraquat-induced oxidative stress (S2C Fig). The deleterious consequences of wild type SOD1 protein overexpression likely complicate analysis of ALS using overexpression models. The second caveat is that overexpression studies are not designed to determine if ALS SOD1 alleles also decrease SOD1 function *in vivo* and thereby contribute to ALS-associated defects. Although ALS SOD1 overexpression models will continue to play a critical role in understanding gain of function mechanisms underlying ALS, we suggest that relying exclusively on these models is not optimal.

There is mounting evidence that SOD1 loss of function contributes to ALS-associated pathology. Studies in *Drosophila* and mouse suggest that SOD1 loss of function contributes to defects in SOD1 ALS models [19,30]. Our results with *C*. *elegans* models support this; both SOD1 gain and loss of function contribute to defects. Clearly *C*. *elegans sod-1* loss of function renders glutamatergic neurons hypersensitive to oxidative stress, leading to degeneration and death. By contrast, SOD1 gain of function is the predominant driver of cholinergic motor neuron loss after oxidative stress; neurons are lost in both overexpression and single-copy/knock-in model animals. However, oxidative stress is still required for cholinergic neuron loss in these models, suggesting decreased *sod-1* activity may also contribute. It is unclear why oxidative stress is required for motor neuron loss in *C*. *elegans* models. There are at least two possibilities: oxidative stress may induce premature aging [49] or mutant ALS SOD1 may impair oxidative stress response by antagonizing normal SOD1 function [28]. In the latter scenario, ALS SOD1 alleles might cause an antimorphic gain of function, in addition to the widely appreciated neomorphic/novel toxic gain of functions. An obvious molecular mechanism for mutant ALS SOD1 to antagonize normal SOD1 function is to drive misfolding and/or sequestration of normal SOD1 into aggregates. Potential antimorphic SOD1 gain of function is supported by evidence that increasing wild type SOD1 levels can exacerbate defects caused by mutant ALS SOD1 in other model systems [19,48], that expressing human SOD1G85R in wild type *C*. *elegans* causes hypersensitivity to oxidative stress (S2C Fig), and that expression of human SOD1G85R in *C*. *elegans* glutamatergic neurons results in glutamatergic neuron degeneration after oxidative stress (Fig 5H).

Is there a direct mapping of neurotransmitter-subtype sensitivity to different *sod-1* disease alleles between human ALS patients and C. *elegans* knock-in models? This remains unclear for two reasons: human genetic diversity and cross-species conservation. First, A4V is the most common SOD1 disease allele in the United States patient population. Because of the large A4V patient population, one can be confident that this allele usually leads to cholinergic spinal motor neuron degeneration and loss, with characteristic sparing of glutamatergic upper motor neurons. The *C*. *elegans sod-1* A4V model reproduces this neurotransmitter-subtype sensitivity, as only cholinergic motor neurons are lost after oxidative stress, not glutamatergic neurons. By contrast, the other ALS patient alleles examined here are much less frequent in the patient population, with H71Y reported in only 1 family [50] and L84V in two patients [50–52]. Human populations are genetically diverse and we expect that this diversity will impact many aspects of ALS, including which neurons are predominantly affected in a given individual or family. Consequently, we are unable to cross-examine disease severity and progression in patients carrying different SOD1 mutations. And, ALS may be polygenic in some patients, increasing the challenge of ascribing specific defects to specific patient alleles. Indeed, previous studies on ALS SOD1 enzymatic activity failed to show a direct link between loss of enzymatic function and disease severity [53,54]. Additionally, in the patient populations, differences in lifestyle and environmental exposure may also impact ALS. By contrast, *C*. *elegans* and other model organisms are relatively isogenic and live in homogeneous environments, which may facilitate studies that directly compare disease alleles. Finally, in patients, ALS impacts neurons in mid-life suggesting that aging or accumulated damage may contribute to disease. Results presented here focus on stress induced degeneration in young adult animals; we have not yet explored how aging might influence neuronal susceptibility to degeneration in these models. The combined efforts of patients, human geneticists, clinicians, researchers using model organisms, and scientists using other approaches will be required to untangle the connections between different SOD1 ALS alleles, neuronal populations affected in the corresponding patients, and the genetic background in diverse patient populations.

Results presented herein suggest that an underlying premise of the ALS field–that identical pathological mechanisms lead to degeneration of cholinergic and glutamatergic neurons–should perhaps be reconsidered. Mechanisms contributing to glutamatergic and cholinergic neurons may not be identical. The differential susceptibility of cholinergic and glutamatergic neurons in *C*. *elegans* single-copy/knock-in *sod-1* models suggests that 1) decreased *sod-1* function may be more deleterious for glutamatergic neurons and 2) gain of function may be the major contributor to cholinergic neuron degeneration. To our knowledge, this hypothesis has not been previously explored and it may shed light on the connections between ALS and Frontotemporal Dementia (FTD). There is considerable genetic and pathological overlap between these diseases [55], but it remains unclear why specific genes are associated only with ALS, only with FTD, or associated with both diseases. Exploring the hypothesis that mechanisms underlying glutamatergic neurodegeneration are distinct from mechanisms underlying cholinergic neurodegeneration may be useful in delineating and dissecting the pathological pathways that underlie these devastating diseases.

## Materials & methods

### MosSCI mediated *sod-1* knock-in

#### Cloning

A 4391 bp fragment spanning the endogenous wild-type *sod-1* gene, including the *sod-1* promoter, introns and UTRs, was amplified from wild type N2 genomic DNA with primers sodF and *sod-1*RC using the Roche Expand Long Range Kit (SN:04829034001). The resulting *sod-1* fragment was subcloned into the pPD#49.26 vector with NheI+XmaI and mutagenized using the QuickChange II Site-Directed Mutagenesis Kit with primer pairs specific for A4V, H71Y and G85R. These primers are listed under S1 Table. Each mutagenized *sod-1* fragment was excised from the corresponding pPD#49.26 plasmid with NheI+XmaI, and ligated into the pCFJ178 Mos vector with AvrII+XmaI to generate final constructs pHA#725 *sod-1p*::*sod-1A4V*^*M*^, *unc-119(+)*; pHA#702 *sod-1p*::*sod-1H71Y*^*M*^, *unc-119(+)* and pHA724 *sod-1p*::*sod-1G85R*^*M*^, *unc-119(+)*. The wild type *sod-1* fragment was cloned directly into the pCFJ178 Mos vector following PCR amplification and NheI+XmaI digestion to generate pHA#720 *sod-1p*::*sod-1WT*^*M*^, *unc-119(+)*. Another plasmid carrying *unc-119(+)* alone, pHA#723 *unc-119(+)*, was generated as “empty” negative control.

#### Strain construction

The wild type *sod-1* allele, ALS mutant *sod-1* alleles and the empty control carrying *unc-119(+)* rescue alone was integrated into chromosome IV using the Mos1/transposase-mediated homologous recombination system (Frøkjær-Jensen et al., 2009). The final constructs pHA#720 *sod-1p*::*sod-1WT*^*M*^, *unc-119(+)*; pHA#725 *sod-1p*::*sod-1A4V*^*M*^, *unc-119(+)*; pHA#702 *sod-1p*::*sod-1H71Y*^*M*^, *unc-119(+)*; pHA#724 *sod-1p*::*sod-1G85R*^*M*^, *unc-119(+)* and pHA#723 *unc-119(+)* were injected at 50 ng/ml with the standard MosSCI cocktail into EG6700 (*unc-119(ed3)III*; *cxTi10882 IV)* animals along with co-injection markers. Single-copy insertion events were detected by rescue of *unc-119(ed3)* in non-fluorescent animals, and confirmed by PCR genotyping and sequencing. All strains were backcrossed at least four times. The endogenous *unc-119(ed3)* allele was PCR amplified and sequenced to check the replacement of *unc-119(ed3)* with wild type *unc-119(+)* in all strains. Transgenic animals were subsequently crossed into the *sod-1(tm776)* null background to generate single-copy insertion models homozygous for wild-type *sod-1*, mutant *sod-1* and for *sod-1* deletion.

### CRISPR/Cas9-mediated *sod-1* knock-in

#### Cloning

A U6 promoter-driven *sod-1* guide RNA was amplified from the *pU6*::*klp-12*::*sgRNA* (Addgene plasmid # 46170) [56] vector template by replacing the *klp-12* targeting sequence with *sod-1* specific primer pair *sod-1_guide2_f* and *sod-1_guide2_r*. The resulting DNA was circularized to generate the final construct pHA0816 *pU6*::*sod-1*::*guide2*.

#### Strain construction

*sod-1* alleles *L84V*^*C*^, *G85R*^*C*^ and *G93A*^*C*^ were introduced into the endogenous *sod-1* locus on chromosome II using CRISPR/Cas9-mediated oligo-templated homologous recombination and *pha-1(ts)* co-conversion, using previously published methods [57]. The final guide RNA construct pHA0816 *pU6*::*sod-1*::*guide2* was injected at 50 ng/ul into temperature-sensitive GE24 (*pha-1(e2123) III*) animals with 50 ng/ul of *Peft-3*::*Cas9*, 50ng/ul of pJW1285 *pha-*1 sgRNA, 10 uM of 200mer sense *pha-1(+)* rescue oligo [57] and 10uM of a mutation specific single-stranded oligodeoxynucleotide (ssODN) listed in S1 Table. Additional silent codon changes were introduced to generate a MscI restriction site, and to inactivate the endogenous PAM site in transgenic lines. A wild type *sod-1WT*^*C*^ control was also generated to control for the silent mutations. Putative recombinants among *pha-1(+)* rescued animals were screened for insertion events with PCR amplification using primers sod-1cIIgenoF and sod-1cIIgenoRC1, followed by MscI digestion. Isolated alleles were verified by sequencing and backcrossed four times. The endogenous *pha-1* locus was PCR amplified and sequenced to verify the replacement of repaired *pha-1* allele with wild type *pha-1(+)* in all strains.

### Neuronal death assays

Cholinergic (*unc-17p*::*GFP or cho-1p*::*mCherry*), GABAergic (*unc-47p*::*GFP*), glutamatergic (*osm-10p*::*GFP*), serotonergic (*tph-1p*::*GFP*) and dopaminergic (*dat-1p*::*GFP*) specific neuronal markers were individually crossed into ALS models to assess neuron loss. A full list of strains used in this study can be found in S2 Table. Day 1 adult animals were mounted on 2% (vol/vol) agar pads and immobilized with 30 mg/mL 2-3-butanedione monoxime (BDM, Sigma) in M9 buffer. Fluorescent neurons were visualized and scored at the microscope for cell death based on loss of neuronal GFP under 63x or 100x objectives (Zeiss AxioImager ApoTome and AxioVision software v4.8). For scoring cholinergic (*unc-17p*::*GFP or cho-1p*::*mCherry*) neurons, animals missing at least two neurons were scored as defective. For scoring *unc-47p*::*GFP*, nineteen GABAergic ventral nerve cord motor neurons were scored. For scoring *osm-10p*::*GFP*, only ASH amphid sensory neurons neurons were scored due to variable/faint GFP expression in ASI and PHB neurons. For paraquat trials, animals were exposed to 2.5 mM paraquat overnight on plates.

### Glutamatergic neuron degeneration

Day 1 adult animals were washed off plates with M9 and incubated with DiD (Fisher DilC18(5) D307) in a microfuge tube as in [58]. After 1.5 hours, animals were spun down at 10000 rpm for 1 min, and transferred to a regular NGM plate. After 1 hour, animals were mounted on 2% (vol/vol) agar pads and immobilized with 30 mg/mL 2-3-butanedione monoxime (BDM, Sigma) in M9 buffer. Fluorescent neuronal cell bodies were visualized and scored for lack of dye uptake under 63x or 100x objectives (Zeiss AxioImager ApoTome and AxioVision software v4.8). For paraquat trials, animals were exposed to 2.5 mM paraquat overnight on plates.

### Survival assays

Animal reared under normal culture conditions at 25 ^o^C were scored for survival on alternating days starting from the first day of adulthood. FUDR was omitted from the growth medium as it alters *C*. *elegans* lifespan for some genotypes [59]. To avoid progeny contamination and overcrowding, aging animals were transferred to a new seeded plate every other day until all animals stopped laying eggs. Animals unresponsive to touch were scored as dead. To score survival on paraquat, we prepared fresh 2.5 mM paraquat (Sigma-Aldrich 856177) plates every week. Again, animals were transferred to new paraquat plates every day until all animals stopped laying eggs. In both assays, bagged animals or animals that left the plate were censored; these animals were included in lifespan determinations until the day before censoring.

### Scoring neuronal inclusions

Day 1 adult animals expressing human SOD1WT-YFP were mounted on 2% (vol/vol) agar pads and immobilized with 30 mg/mL 2-3-butanedione monoxime (BDM) (Sigma) in M9 buffer. Animals were quantified for inclusions within the motor neurons along the ventral nerve cord under 63x or 100x objectives (Zeiss AxioImager ApoTome and AxioVision software v4.8). For paraquat trials, animals were exposed to 2.5 mM paraquat for 3 hours on plates, as overnight exposure to paraquat problematically increased background fluorescence.

### Aldicarb resistance sssay

Animals reared at 25 ^o^C were scored for paralysis on 1 mM aldicarb (Sigma-Aldrich 33386) over the course of 7 hours. NGM plates containing 1 mM aldicarb were freshly poured the day before the assay. Aldicarb plates were seeded with 30 ul of OP50, and left to dry open-lid under the hood for 30 minutes. Day 1 adult animals were then transferred onto aldicarb plates, and scored for paralysis every hour. Aldicarb-induced paralysis was scored as inability to move/pump to sequential prodding with a metal wire twice in the tail and then twice in the head. Paralyzed animals were removed from the plate and not re-counted.

### Synaptic protein puncta analysis

For synaptic puncta imaging, day 1 adult animals were mounted on 2% (vol/vol) agar pads and immobilized using 30 mg/mL 2-3-butanedione monoxime (BDM) (Sigma) in M9 buffer. Images were captured in z-stacks from dorsal cord posterior to vulva (100x objective, Zeiss AxioImager ApoTome and AxioVision software v4.8). Data from three independent trials (*n* > 20 animals in total/genotype) was analyzed. Puncta total intensity, width, and linear density were quantified using the Punctaanalyser program in Matlab (v6.5; Mathworks, Inc., Natick, MA, USA; RRID:SCR_001622) [60].

### Behavioral assays

#### Nose-touch avoidance

Nose touch assays were performed as described elsewhere [39]. Animals were exposed to 2.5 mM paraquat on plates for 4 hours, as overnight exposure dramatically perturbs nose touch response in wild type/N2 animals. Percent trials in which animals responded to touch by stopping forward movement or initiating reverse movement is reported.

#### Locomotion

Day 1 adult animals treated overnight with 2.5 mM paraquat were transferred into 60 ul of M9 buffer pipetted into a 1 mm diameter ring on a microscope slide. Videos were recorded at 18 frames per second for 30 seconds, and analyzed with computer vision software CeleST [31].

### Statistical analysis

Data collection and analysis were performed by experimenters blinded to genotype and, when possible, treatment. Quantitative data was analyzed using Graph Pad Prism 6 software (La Jolla, CA). Statistical significance for the survival and aldicarb resistance assays was determined with log-rank test. Kruskal-Wallis test was used to determine statistical significance for the swimming locomotion assays. For the remainder of the assays, two-tailed t-test or chi-square test was used to determine significance. A value of *P* < 0.05 was used to establish statistical significance. Error bars in figures represent error of the mean (S.E.M.).

## Supporting information

S1 Fig Lifespan studies using ALS *sod-1* models. **(A and B)** To assess lifespan in *C*. *elegans* knock-in models, we scored survival at 25 ^o^C on alternating days from pre-adulthood (L4 stage) until death. Single-copy *sod-1G85R*^*M*^ (Panel A) and *sod-1G85R*^*C*^ (Panel B) lifespan did not differ from those of appropriate wild type controls (*sod-1G85R*^*M*^ vs *sod-1WT*^*M*^, *P* = 0.64; *sod-1G85R*^*C*^ vs *sod-1WT*^*C*^, *P* = 0.32). Single-copy/knock-in animals *sod-1A4V*^*M*^, *sod-1H71Y*^*M*^, *sod-1L84V*^*C*^ and *sod-1G93A*^*C*^, as well as animals lacking endogenous *sod-1* or *empty*^*M*^ controls, showed a modest decrease in lifespan, compared to their respective wild type controls. The largest difference in median lifespan was observed in *sod-1H17Y*^*M*^ animals; median lifespan decreased from 14 to 9 days. *sod-1L84V*^*C*^ indicated with #. *sod-1(+)* is standard N2 strain. Survival was scored in the absence of FUDR under standard culture conditions, moving animals to new plates every other day to avoid progeny contamination. Animals that left the plate or animals with internal egg hatching were censored; these animals were included in lifespan determinations until the day before censoring. N was 30 for all genotypes in both panels in each of 3 independent trials. S4 Table summarizes these results from the three independent replicates. Log-rank test: * *P* < 0.05; ** *P* < 0.01; *** *P* < 0.001.
**(C and D)** The impact of neuronal overexpression of human SOD1 on *C*. *elegans* lifespan has not been examined previously. We found that neuronal overexpression of human SOD1G85R (*hSOD1G85R-YFP*^*OE*^) did not decrease survival, relative to *hSDOD1WT-YFP*^*OE*^ controls (*P* = 0.11, Panel C). And, lifespan of animals overexpressing the human wild type SOD1 protein (*hSDOD1WT-YFP*^*OE*^) in neurons was not different than *sod-1(+)* wild type controls (*P* = 0.36, Panel C). However, neuronal overexpression of human wild type SOD1 did not rescue lifespan in *sod-1(-)* animals, which lack the endogenous *sod-1* gene (*P* = 0.44 for *sod-1(-); hSOD1WT-YFP*^*OE*^ vs *sod-1(-)*, Panel D). However, neuronal overexpression of human *SOD1G85R* in *sod-1(-)* background modestly restored lifespan compared to *sod-1(-)* animals; this difference is seen predominantly at midlife. Genotypes, analysis and methods as in Panels A-B, with 30 animals for all genotypes in both panels in each of three independent trials. S4 Table summarizes these results from the three independent replicates.
(TIF)

S2 Fig Assessment of *C*. *elegans* survival under oxidative stress. **(A and B)** To determine the impact of oxidative stress in ALS SOD1/*sod-1* models, we scored survival at 25°C every day from pre-adulthood (L4 stage) in animals exposed to 2.5 mM paraquat. *sod-1H71Y*^*M*^ and *sod-1G85R*^*M*^ animals had decreased survival, compared to *sod-1WT*^*M*^ animals under oxidative stress (Panel A). Loss of *sod-1* in *empty*^*M*^ controls similarly decreased survival, relative to *sod-1WT*^*M*^ wild type control animals. *sod-1A4V*^*M*^ animals had increased survival under oxidative stress, compared to *sod-1WT*^*M*^ controls. *sod-1L84V*^*C*^, *sod-1G85R*^*C*^ and *sod-1G93A*^*C*^ animals had decreased survival compared to *sod-1WT*^*C*^ controls (Panel B). *sod-1L84V*^*C*^ indicated with #. *sod-1(+)* is the standard N2 strain. Survival was scored in the absence of FUDR, moving animals to new plates every other day to avoid progeny contamination. Animals that left the plate or animals with internal egg hatching were censored; these animals were included in survival determinations until the day before censoring. N was 30 for all genotypes in both panels in each one of the three independent trials, except for one trial that was started with 20 *sod-1G85R*^*C*^ animals in Panel B. Supplemental S4 Table summarizes these results from the three independent replicates. Log-rank test: * *P* < 0.05; ** *P* < 0.01; *** *P* < 0.001. *sod-1(-)*: *sod-1(tm776)*.
**(C and D)** Neuronal overexpression of human SOD1G85R-YFP further decreased survival under paraquat-induced oxidative stress compared to *hSOD1WT-YFP*^*OE*^ animals (Panel C). Loss of *sod-1* decreased survival under oxidative stress compared to *sod-1(+)* wild type controls (Panel D). Additionally, oxidative stress sensitivity in *sod-1(-)* animals was partially rescued by neuronal *hSOD1WT-YFP*^*OE*^ (Panel D). Genotypes, analysis and methods as in Panels A-B. N was 30 for all genotypes in both panels in each one of the three independent trials. S4 Table summarizes these results from the three independent replicates.
(TIF)

S3 Fig Presynaptic ITSN-1 and SNB-1 puncta are not altered in single-copy models or in animals lacking *sod-1* function. Perturbations in presynaptic signaling or loss of synapses at the *C*. *elegans* NMJ can be detected as changes in the accumulation of fluorescently-tagged pre-synaptic proteins. We found that GFP-tagged SNB-1 (Panel A) and ITSN-1 (Panel B) accumulation and levels were unperturbed in single-copy *sod-1H71Y*^*M*^ and *sod-1G85R*^*M*^ animals compared to *sod-1WT*^*M*^ controls. Previous work reported that SNB-1::GFP is altered in human SOD1G85R overexpression animals [13]. N > 22 for each genotype from at least two independent trials. Two-tailed t-test.
(TIF)

S4 Fig Neuronal specificity of oxidative stress induced neurodegeneration. **(A)** Serotonergic neurons NSM, ADFL and ADFR (scored using *tph-1p*::*GFP*) were intact in *sod-1G85R*^*C*^ and *sod-1(-)* animals after paraquat treatment. *sod-1(+)* designates the unedited wild type gene at the endogenous locus; this is the same allele present in standard N2 strain. For panels A-C: three independent trials. Error bars indicate ±SEM. N > 30 per genotype. Two-tailed t-test: * *P* < 0.05; ** *P* < 0.01; *** *P* < 0.001.
**(B)** Dopaminergic neurons CEPVL, CEPVR, CEPDL, CEPDR, ADEL, ADER, and ADEL/R sensory processes (scored using *dat-1p*::*GFP*) were intact in *sod-1G85R*^*C*^ and *sod-1(-)* animals after paraquat treatment. *sod-1(+)* as in Panel A.
**(C)** ASH neurons (scored using *osm-10p*::*GFP*) were lost in *sod-1G85R*^*C*^ and *sod-1(-)* animals after paraquat treatment, compared to *sod-1WT*^*C*^ and *sod-1(+)* controls, respectively. PHA and PHB neurons were not scored due to inconsistent PHB GFP expression in wild type controls.
**(D)** GABAergic motor neurons (scored using *unc-47p*::*GFP*) were lost only in the *sod-1G85R*^*M*^ animals compared to *sod-1WT*^*M*^ controls. Three independent trials. N > 20 per genotype. Chi-square test.
**(E)** To examine the consequences of altering gene dosage and assess recessive/dominance of single-copy/knock-in ALS alleles, homozygous ALS *sod-1* model animals and controls were crossed to homozygous *sod-1WT*^*M*^ or *empty*^*M*^ males carrying a GFP-expressing transgene and cross-progeny tested for DiD dye-uptake in ASH neurons after paraquat treatment. *empty*^*M*^/*empty*^*M*^ cross-progeny had defects after paraquat treatment, compared to *sod-1WT*^*M*^*/sod-1WT*^*M*^ cross-progeny, while *sod-1WT*^*M*^*/empty*^*M*^ cross-progeny had intact glutamatergic neurons. *sod-1H71Y*^*M*^*/empty*^*M*^ and *sod-1G85R*^*M*^*/empty*^*M*^ animals were defective compared to *sod-1WT*^*M*^*/empty*^*M*^ animals. Conversely, *sod-1WT*^*M*^*/sod-1H71Y*^*M*^ or *sod-1WT*^*M*^/*sod-1G85R*^*M*^ animals had intact glutamatergic neurons after paraquat treatment. Three independent trials. N > 25 per genotype. Error bars indicate ±SEM. Two-tailed t-test: * *P* < 0.05; ** *P* < 0.01; *** *P* < 0.001.
(TIF)

S1 Table Primers used in this study. (PDF)

S2 Table Strains used in this study. (PDF)

S3 Table ALS SOD1 disease mutations in the human protein and their equivalent locations in the *C*. *elegans* SOD-1 protein. (PDF)

S4 Table Survival data from independent replicates relating to S1 Fig and S2 Fig. MST, Median Survival Time; n is the number of total subjects at the beginning of each trial and x is the total number of subjects censored by the end of the study; R stands for replica; Avg MST is the average of the three individual MST determinations; Pooled MST was calculated by pooling data from all replicates; SEM, standard error of the median; Und, undefined due to high number of censored subjects. In S1 Fig, we scored survival at 25 ^o^C on alternating days from pre-adulthood (L4 stage) until death. In S2 Fig, we scored survival at 25 ^o^C every day from pre-adulthood (L4 stage) in animals exposed to 2.5 mM paraquat. *sod-1(+)* is the standard N2 strain. Survival was scored in the absence of FUDR. Animals were transferred to new plates every other day to avoid progeny contamination. Animals that left the plate or animals with internal egg hatching were censored; these animals were included in lifespan determinations until the day before censoring. N was 30 for all genotypes in all panels in each of 3 independent trials, except for *sod-1G85R*^*C*^ in “+” paraquat R1 trial which had 20 animals (S2B Fig). MST and log-rank test significance was determined using Graph Pad Prism 6 software (La Jolla, CA).
(PDF)

S5 Table Summary of the results presented in Figs 2–5 and S1 and S2 Figs. Directly comparable data sets are flanked by double lines. Appropriate controls are indicated within each experimental set. Genotypes with increases and decreases are indicated with a shade of red and blue, respectively. n.s., not significant; n.a., not available.
(PDF)
