# Supplementary figures and images for: Single copy/knock-in models of ALS SOD1 in C. elegans suggest loss and gain of function have different contributions to cholinergic and glutamatergic neurodegeneration
Source: PLoS Genet. 2018 Oct 8;14(10):e1007682. doi: 10.1371/journal.pgen.1007682 (PMC6200258; doi:10.1371/journal.pgen.1007682)

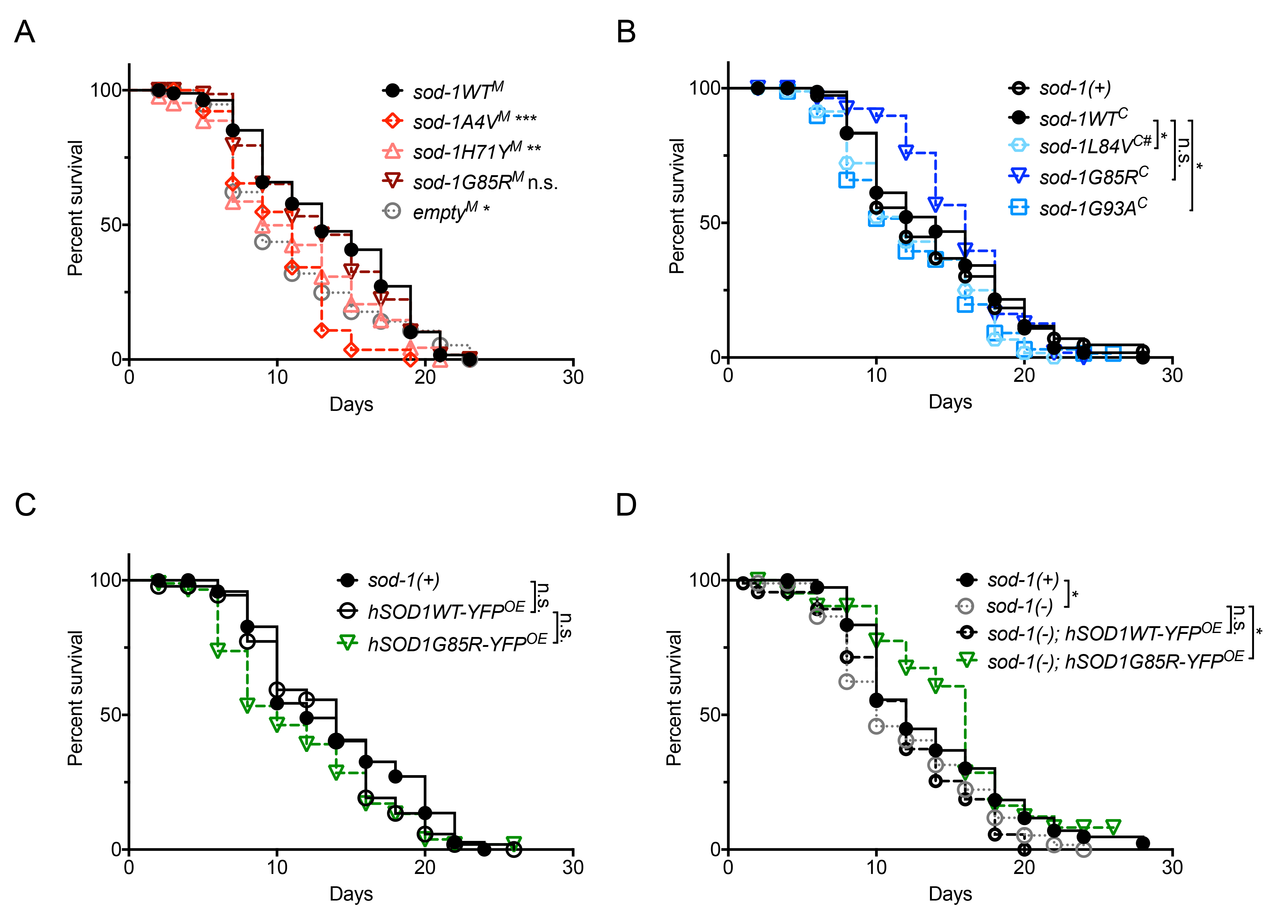

Supplement: S1 Fig — (A and B) To assess lifespan in C. elegans knock-in models, we scored survival at 25 oC on alternating days from pre-adulthood (L4 stage) until death. Single-copy sod-1G85RM (Panel A) and sod-1G85RC (Panel B) lifespan did not differ from those of appropriate wild type controls (sod-1G85RM vs sod-1WTM, P = 0.64; sod-1G85RC vs sod-1WTC, P = 0.32). Single-copy/knock-in animals sod-1A4VM, sod-1H71YM, sod-1L84VC and sod-1G93AC, as well as animals lacking endogenous sod-1 or emptyM controls, showed a modest decrease in lifespan, compared to their respective wild type controls. The largest difference in median lifespan was observed in sod-1H17YM animals; median lifespan decreased from 14 to 9 days. sod-1L84VC indicated with #. sod-1(+) is standard N2 strain. Survival was scored in the absence of FUDR under standard culture conditions, moving animals to new plates every other day to avoid progeny contamination. Animals that left the plate or animals with internal egg hatching were censored; these animals were included in lifespan determinations until the day before censoring. N was 30 for all genotypes in both panels in each of 3 independent trials. S4 Table summarizes these results from the three independent replicates. Log-rank test: * P < 0.05; ** P < 0.01; *** P < 0.001. (C and D) The impact of neuronal overexpression of human SOD1 on C. elegans lifespan has not been examined previously. We found that neuronal overexpression of human SOD1G85R (hSOD1G85R-YFPOE) did not decrease survival, relative to hSDOD1WT-YFPOE controls (P = 0.11, Panel C). And, lifespan of animals overexpressing the human wild type SOD1 protein (hSDOD1WT-YFPOE) in neurons was not different than sod-1(+) wild type controls (P = 0.36, Panel C). However, neuronal overexpression of human wild type SOD1 did not rescue lifespan in sod-1(-) animals, which lack the endogenous sod-1 gene (P = 0.44 for sod-1(-); hSOD1WT-YFPOE vs sod-1(-), Panel D). However, neuronal overexpression of human SOD1G85R in sod-1(-) [file pgen.1007682.s001.tif]

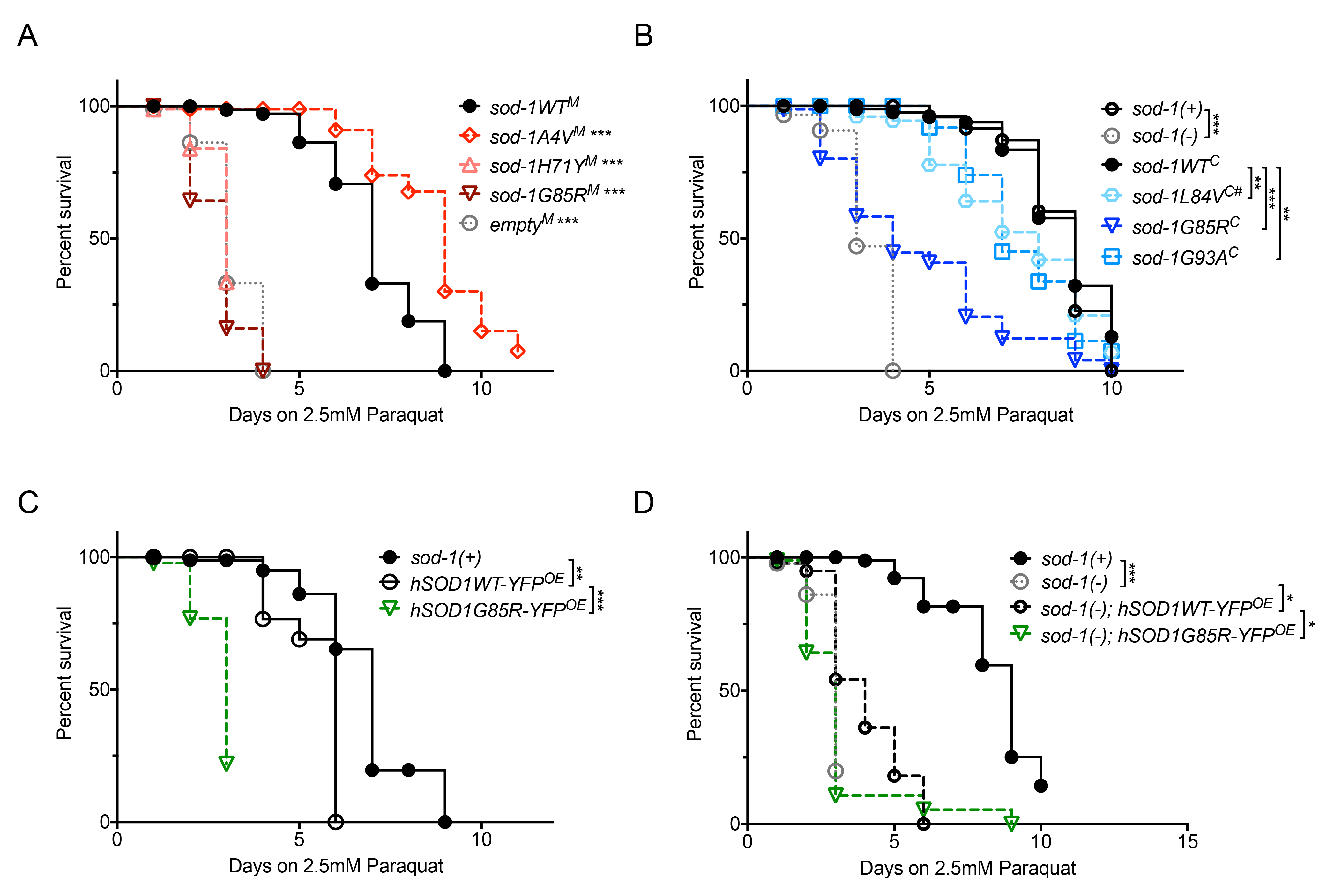

Supplement: S2 Fig — (A and B) To determine the impact of oxidative stress in ALS SOD1/sod-1 models, we scored survival at 25°C every day from pre-adulthood (L4 stage) in animals exposed to 2.5 mM paraquat. sod-1H71YM and sod-1G85RM animals had decreased survival, compared to sod-1WTM animals under oxidative stress (Panel A). Loss of sod-1 in emptyM controls similarly decreased survival, relative to sod-1WTM wild type control animals. sod-1A4VM animals had increased survival under oxidative stress, compared to sod-1WTM controls. sod-1L84VC, sod-1G85RC and sod-1G93AC animals had decreased survival compared to sod-1WTC controls (Panel B). sod-1L84VC indicated with #. sod-1(+) is the standard N2 strain. Survival was scored in the absence of FUDR, moving animals to new plates every other day to avoid progeny contamination. Animals that left the plate or animals with internal egg hatching were censored; these animals were included in survival determinations until the day before censoring. N was 30 for all genotypes in both panels in each one of the three independent trials, except for one trial that was started with 20 sod-1G85RC animals in Panel B. Supplemental S4 Table summarizes these results from the three independent replicates. Log-rank test: * P < 0.05; ** P < 0.01; *** P < 0.001. sod-1(-): sod-1(tm776). (C and D) Neuronal overexpression of human SOD1G85R-YFP further decreased survival under paraquat-induced oxidative stress compared to hSOD1WT-YFPOE animals (Panel C). Loss of sod-1 decreased survival under oxidative stress compared to sod-1(+) wild type controls (Panel D). Additionally, oxidative stress sensitivity in sod-1(-) animals was partially rescued by neuronal hSOD1WT-YFPOE (Panel D). Genotypes, analysis and methods as in Panels A-B. N was 30 for all genotypes in both panels in each one of the three independent trials. S4 Table summarizes these results from the three independent replicates. (TIF) [file pgen.1007682.s002.tif]

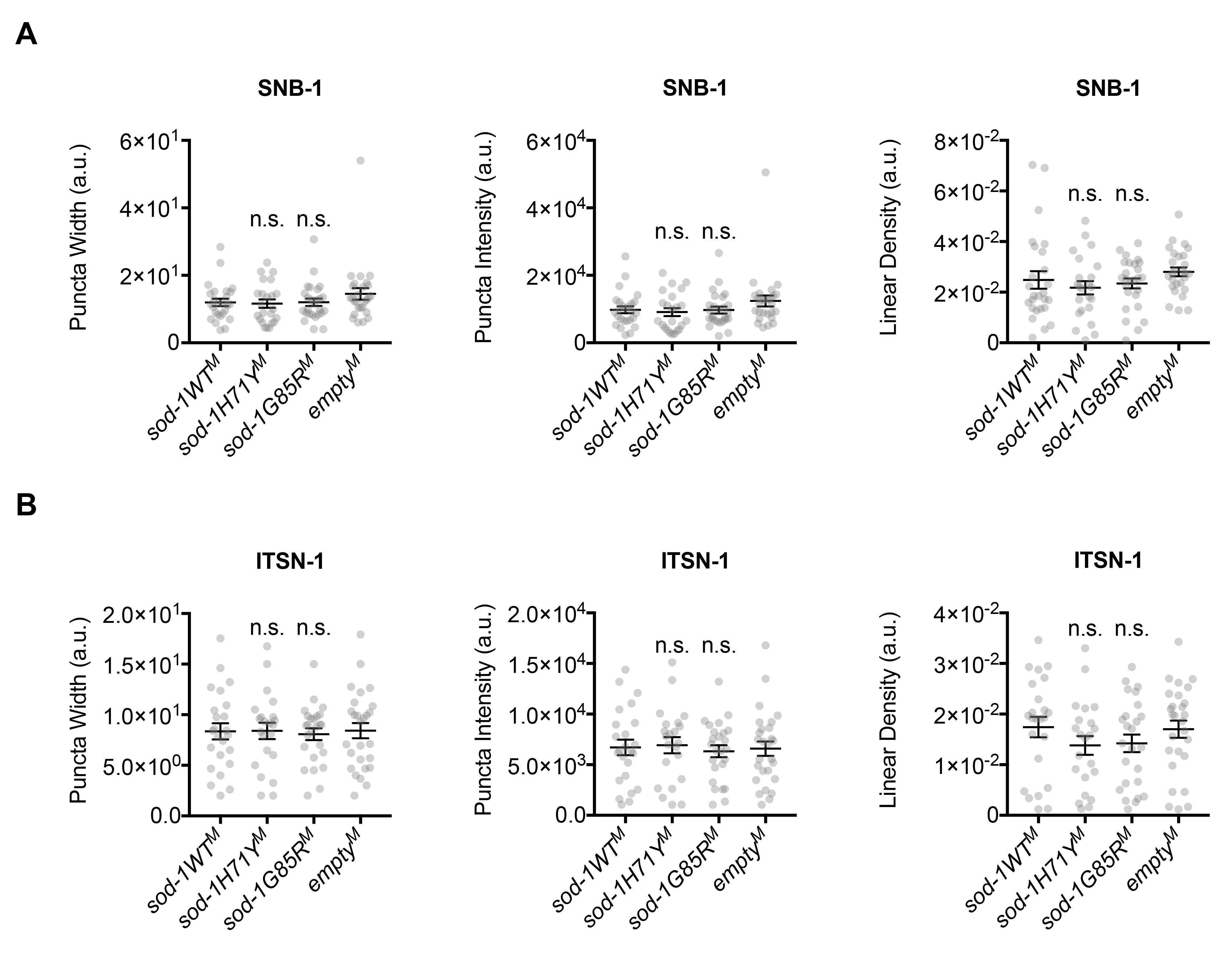

Supplement: S3 Fig — Perturbations in presynaptic signaling or loss of synapses at the C. elegans NMJ can be detected as changes in the accumulation of fluorescently-tagged pre-synaptic proteins. We found that GFP-tagged SNB-1 (Panel A) and ITSN-1 (Panel B) accumulation and levels were unperturbed in single-copy sod-1H71YM and sod-1G85RM animals compared to sod-1WTM controls. Previous work reported that SNB-1::GFP is altered in human SOD1G85R overexpression animals [13]. N > 22 for each genotype from at least two independent trials. Two-tailed t-test. (TIF) [file pgen.1007682.s003.tif]

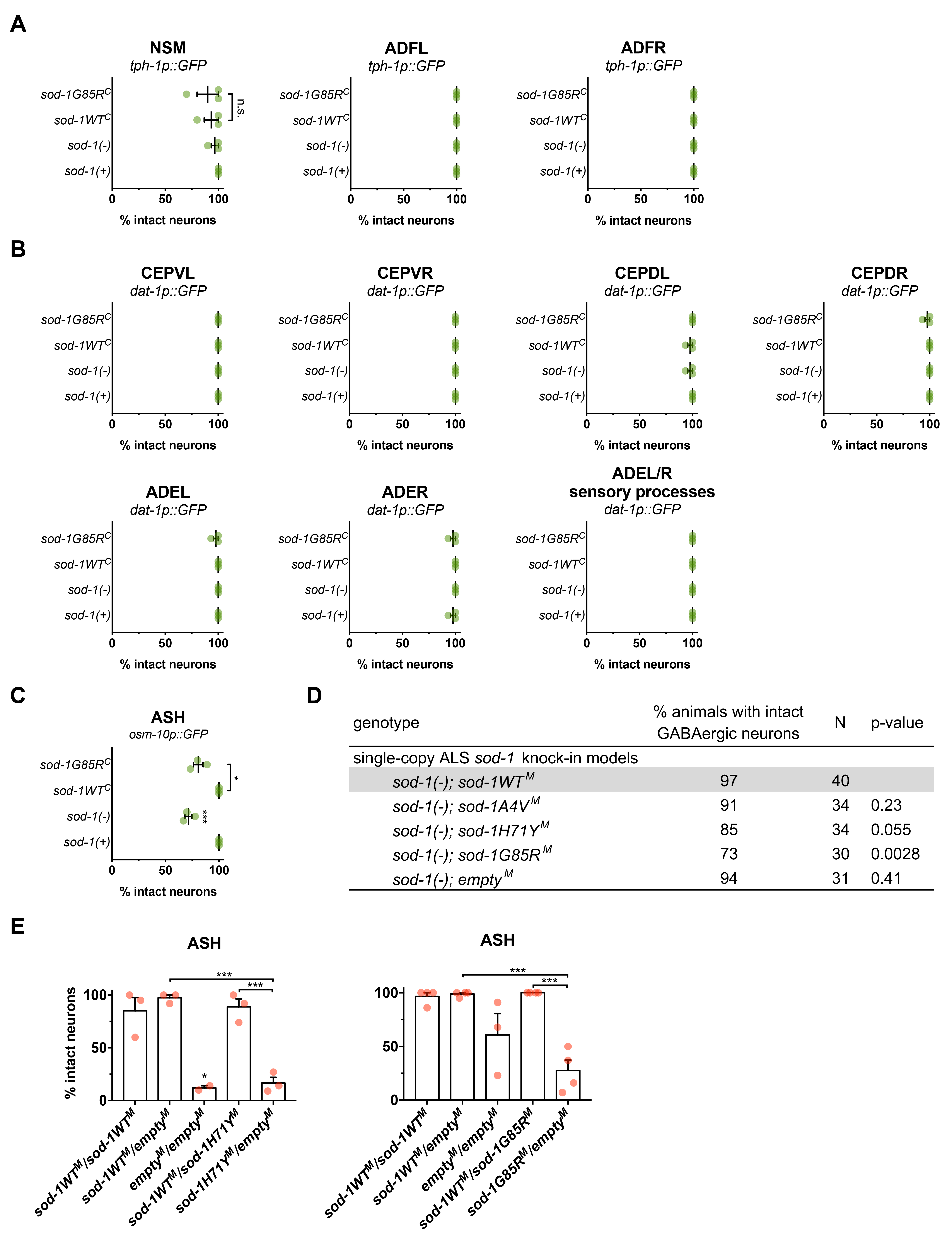

Supplement: S4 Fig — (A) Serotonergic neurons NSM, ADFL and ADFR (scored using tph-1p::GFP) were intact in sod-1G85RC and sod-1(-) animals after paraquat treatment. sod-1(+) designates the unedited wild type gene at the endogenous locus; this is the same allele present in standard N2 strain. For panels A-C: three independent trials. Error bars indicate ±SEM. N > 30 per genotype. Two-tailed t-test: * P < 0.05; ** P < 0.01; *** P < 0.001. (B) Dopaminergic neurons CEPVL, CEPVR, CEPDL, CEPDR, ADEL, ADER, and ADEL/R sensory processes (scored using dat-1p::GFP) were intact in sod-1G85RC and sod-1(-) animals after paraquat treatment. sod-1(+) as in Panel A. (C) ASH neurons (scored using osm-10p::GFP) were lost in sod-1G85RC and sod-1(-) animals after paraquat treatment, compared to sod-1WTC and sod-1(+) controls, respectively. PHA and PHB neurons were not scored due to inconsistent PHB GFP expression in wild type controls. (D) GABAergic motor neurons (scored using unc-47p::GFP) were lost only in the sod-1G85RM animals compared to sod-1WTM controls. Three independent trials. N > 20 per genotype. Chi-square test. (E) To examine the consequences of altering gene dosage and assess recessive/dominance of single-copy/knock-in ALS alleles, homozygous ALS sod-1 model animals and controls were crossed to homozygous sod-1WTM or emptyM males carrying a GFP-expressing transgene and cross-progeny tested for DiD dye-uptake in ASH neurons after paraquat treatment. emptyM/emptyM cross-progeny had defects after paraquat treatment, compared to sod-1WTM/sod-1WTM cross-progeny, while sod-1WTM/emptyM cross-progeny had intact glutamatergic neurons. sod-1H71YM/emptyM and sod-1G85RM/emptyM animals were defective compared to sod-1WTM/emptyM animals. Conversely, sod-1WTM/sod-1H71YM or sod-1WTM/sod-1G85RM animals had intact glutamatergic neurons after paraquat treatment. Three independent trials. N > 25 per genotype. Error bars indicate ±SEM. Two-tailed t-test: * P < 0.05; ** P < 0.01; *** P < 0.001. (TIF) [file pgen.1007682.s004.tif]
