# Supplementary material for: Single copy/knock-in models of ALS SOD1 in C. elegans suggest loss and gain of function have different contributions to cholinergic and glutamatergic neurodegeneration
Source: PLoS Genet. 2018 Oct 8;14(10):e1007682. doi: 10.1371/journal.pgen.1007682 (PMC6200258; doi:10.1371/journal.pgen.1007682)
Supplement: S1 Table — (PDF) [file pgen.1007682.s005.pdf]

**Primer Name**	**Sequence**
sodF	tgatgctagcggaaggaacagacgttttgc
sod-1RC	tgatcccgggtggctctattcagaaagaaacg
sod1A4V-F	ctcaaaaaatgtcgaaccgtgttgtcgctgttcttcgtgg
sod1A4V-RC	ccacgaagaacagcgacaacacggttcgacatttttgag
sod1H71Y-F	caatccatttggaaagacttatggtggaccaaaagtatgg
sod1H71Y-RC	ccatacttttggtccaccataagtctttccaaatggattg
sod1G85R-F	tcacgtaggcgatctacgaaatgtggaagctggagc
sod1G85R-RC	gctccagcttccacatttcgtagatcgcctacgtga
sod-1_guide2_f	acgagcgtgtgttttagagctagaaatagc
sod-1_guide2_r	cacgctttacaaacatttagatttgcaattc
sod-1WT_ssODN	cggccaacgacagtgtttggaccgtaaagcgtgacgagcgtgtccgtgagcttgattttggccactccatcggctccagc ttccacatttcctagatcgcctacgtg
sod-1L84V_ssODN	cggccaacgacagtgtttggaccgtaaagcgtgacgagcgtgtccgtgagcttgattttggccactccatcggctccagc ttccacatttcctacatcgcctacgtgacggatctcggactgaaaaaatattataattc
sod-1G85R_ssODN	cggccaacgacagtgtttggaccgtaaagcgtgacgagcgtgtccgtgagcttgattttggccactccatcggctccagc ttccacatttcgtagatcgcctacgtgacggatctcggactgaaaaaatattataattc
sod-1G93A_ssODN	cggccaacgacagtgtttggaccgtaaagcgtgacgagcgtgtccgtgagcttgattttggccacagcatcggctccag cttccacatttcctagatcgcctacgtg
sod-1cIIgenoF	aaacttttctgggcaattcg
sod-1cIIgenoRC1	gcatctatcgactgtgatctgc
pha-1_200_ssODN	ggagttttgttgtacattacatttcaggttcttaaaacaaaccatgaagattatggtaatcaaaatacgaatcgaagactca aaaagagtatgctgtatgattacagatgttcatcaagttattcataaatcattgataggttcagattgtaagtcttgattatctat cgttttgtaaagtgactaaactttaatcatta
pha-1_geno_f	caatttggcagccattcatgtg
pha-1_geno_r	tcgcgcactactgaatcagagtc
