# Supplementary material for: Single copy/knock-in models of ALS SOD1 in C. elegans suggest loss and gain of function have different contributions to cholinergic and glutamatergic neurodegeneration
Source: PLoS Genet. 2018 Oct 8;14(10):e1007682. doi: 10.1371/journal.pgen.1007682 (PMC6200258; doi:10.1371/journal.pgen.1007682)
Supplement: S2 Table — (PDF) [file pgen.1007682.s006.pdf]

Strain name	Genotype
N2	sod-1(+) II
HA2281	sod-1(tm776) II
EG6700	unc-119(ed3) III; cxTi10882 IV; oxEx1579
VS21	hjSi20 [myo-2p::mCherry::unc-54 3'UTR] IV
HA2395	sod-1(tm776) II; unc-119(+) III; hjSi20 [myo-2p::mCherry::unc-54 3'UTR] IV; him-5 V
HA2297	sod-1(+) II; unc-119(+) III; rtSi001 [sod-1p::sod-1WT^{M}::sod-1 3'UTR + Cbr-unc-119(+)] IV
HA2408	sod-1(+) II; unc-119(+) III; rtSi008 [sod-1p::sod-1A4V^{M}::sod-1 3'UTR + Cbr-unc-119(+)] IV
HA2309	sod-1(+) II; unc-119(+) III; rtSi007 [sod-1p::sod-1H71Y^{M}::sod-1 3'UTR + Cbr-unc-119(+)] IV
HA2308	sod-1(+) II; unc-119(+) III; rtSi006 [sod-1p::sod-1G85R^{M}::sod-1 3'UTR + Cbr-unc-119(+)] IV
HA2621	sod-1(+) II; unc-119(+) III; rtSi026 [Cbr-unc-119(+)] IV
HA2619	sod-1(tm776) II; unc-119(+) III; rtSi001 [sod-1p::sod-1WT^{M}::sod-1 3'UTR + Cbr-unc-119(+)] IV
HA2464	sod-1(tm776) II; unc-119(+) III; rtSi008 [sod-1p::sod-1A4V^{M}::sod-1 3'UTR + Cbr-unc-119(+)] IV
HA2425	sod-1(tm776) II; unc-119(+) III; rtSi007 [sod-1p::sod-1H71Y^{M}::sod-1 3'UTR + Cbr-unc-119(+)] IV
HA2426	sod-1(tm776) II; unc-119(+) III; rtSi006 [sod-1p::sod-1G85R^{M}::sod-1 3'UTR + Cbr-unc-119(+)] IV
HA2622	sod-1(tm776) II; unc-119(+) III; rtSi026 [Cbr-unc-119(+)] IV
HA2532	sod-1(tm776) II; unc-119(+) III; rtSi001 [sod-1p::sod-1WT^{M}::sod-1 3'UTR + Cbr-unc-119(+)] IV; oxIs12 [unc-47p::GFP] X
HA2533	sod-1(tm776) II; unc-119(+) III; rtSi008 [sod-1p::sod-1A4V^{M}::sod-1 3'UTR + Cbr-unc-119(+)] IV; oxIs12 [unc-47p::GFP] X
HA2534	sod-1(tm776) II; unc-119(+) III; rtSi007 [sod-1p::sod-1H71Y^{M}::sod-1 3'UTR + Cbr-unc-119(+)] IV; oxIs12 [unc-47p::GFP] X
HA2535	sod-1(tm776) II; unc-119(+) III; rtSi006 [sod-1p::sod-1G85R^{M}::sod-1 3'UTR + Cbr-unc-119(+)] IV; oxIs12 [unc-47p::GFP] X
HA2679	sod-1(tm776) II; unc-119(+) III; rtSi026 [Cbr-unc-119(+)] IV; oxIs12 [unc-47p::GFP] X
HA2615	sod-1(tm776) II; unc-119(+) III; rtSi001 [sod-1p::sod-1WT^{M}::sod-1 3'UTR + Cbr-unc-119(+)] IV; [snb-1p::hSOD1WT::YFP]
HA2620	sod-1(tm776) II; unc-119(+) III; rtSi008 [sod-1p::sod-1A4V^{M}::sod-1 3'UTR + Cbr-unc-119(+)] IV; [snb-1p::hSOD1WT::YFP]
HA2617	sod-1(tm776) II; unc-119(+) III; rtSi007 [sod-1p::sod-1H71Y^{M}::sod-1 3'UTR + Cbr-unc-119(+)] IV; [snb-1p::hSOD1WT::YFP]
HA2616	sod-1(tm776) II; unc-119(+) III; rtSi006 [sod-1p::sod-1G85R^{M}::sod-1 3'UTR + Cbr-unc-119(+)] IV; [snb-1p::hSOD1WT::YFP]
HA2703	sod-1(tm776) II; unc-119(+) III; rtSi026 [Cbr-unc-119(+)] IV; [snb-1p::hSOD1WT::YFP]
LX929	sod-1(+) II; vsIs48 [unc-17p::GFP]
HA3525	sod-1(tm776) II; vsIs48 [unc-17p::GFP]
HA2630	sod-1(tm776) II; unc-119(+) III; rtSi001 [sod-1p::sod-1WT^{M}::sod-1 3'UTR + Cbr-unc-119(+)] IV; vsIs48 [unc-17p::GFP]
HA2631	sod-1(tm776) II; unc-119(+) III; rtSi008 [sod-1p::sod-1A4V^{M}::sod-1 3'UTR + Cbr-unc-119(+)] IV; vsIs48 [unc-17p::GFP]
HA2632	sod-1(tm776) II; unc-119(+) III; rtSi007 [sod-1p::sod-1H71Y^{M}::sod-1 3'UTR + Cbr-unc-119(+)] IV; vsIs48 [unc-17p::GFP]
HA2633	sod-1(tm776) II; unc-119(+) III; rtSi006 [sod-1p::sod-1G85R^{M}::sod-1 3'UTR + Cbr-unc-119(+)] IV; vsIs48 [unc-17p::GFP]
HA2680	sod-1(tm776) II; unc-119(+) III; rtSi026 [Cbr-unc-119(+)] IV; vsIs48 [unc-17p::GFP]
HA2696	sod-1(+) II; unc-119(+) III; rtSi001 [sod-1p::sod-1WT^{M}::sod-1 3'UTR + Cbr-unc-119(+)] IV; vsIs48 [unc-17p::GFP]
HA2695	sod-1(+) II; unc-119(+) III; rtSi008 [sod-1p::sod-1A4V^{M}::sod-1 3'UTR + Cbr-unc-119(+)] IV; vsIs48 [unc-17p::GFP]
HA2701	sod-1(+) II; unc-119(+) III; rtSi007 [sod-1p::sod-1H71Y^{M}::sod-1 3'UTR + Cbr-unc-119(+)] IV; vsIs48 [unc-17p::GFP]
HA2702	sod-1(+) II; unc-119(+) III; rtSi006 [sod-1p::sod-1G85R^{M}::sod-1 3'UTR + Cbr-unc-119(+)] IV; vsIs48 [unc-17p::GFP]
HA2697	sod-1(+) II; unc-119(+) III; rtSi026 [Cbr-unc-119(+)] IV; vsIs48 [unc-17p::GFP]
HA3526	sod-1(tm776) II; [snb-1p::hSOD1WT-YFP]
HA3527	sod-1(tm776) II; [snb-1p::hSOD1G85R-YFP]
HA3378	sod-1(+) II; [snb-1p::hSOD1WT-YFP]; otIs544 [cho-1(fosmid)::SL2::mCherry::H2B + pha-1(+)]
HA3380	sod-1(tm776) II; [snb-1p::hSOD1WT-YFP]; otIs544 [cho-1(fosmid)::SL2::mCherry::H2B + pha-1(+)]
HA3379	sod-1(+) II; [snb-1p::hSOD1G85R-YFP]; otIs544 [cho-1(fosmid)::SL2::mCherry::H2B + pha-1(+)]
HA3381	sod-1(tm776) II; [snb-1p::hSOD1G85R-YFP]; otIs544 [cho-1(fosmid)::SL2::mCherry::H2B + pha-1(+)]
HA3528	sod-1(tm776) II; unc-119(+) III; rtSi001 [sod-1p::sod-1WT^{M}::sod-1 3'UTR + Cbr-unc-119(+)] IV; nuIs175 [myo-2p::RFP, unc-129p::RFP::snb-1] X
HA3529	sod-1(tm776) II; unc-119(+) III; rtSi007 [sod-1p::sod-1H71Y^{M}::sod-1 3'UTR + Cbr-unc-119(+)] IV; nuIs175 [myo-2p::RFP, unc-129p::RFP::snb-1] X
HA3530	sod-1(tm776) II; unc-119(+) III; rtSi006 [sod-1p::sod-1G85R^{M}::sod-1 3'UTR + Cbr-unc-119(+)] IV; nuIs175 [myo-2p::RFP, unc-129p::RFP::snb-1] X
HA3531	sod-1(tm776) II; unc-119(+) III; rtSi026 [Cbr-unc-119(+)] IV; nuIs175 [myo-2p::RFP, unc-129p::RFP::snb-1] X
HA3532	sod-1(tm776) II; unc-119(+) III; rtSi001 [sod-1p::sod-1WT^{M}::sod-1 3'UTR + Cbr-unc-119(+)] IV; nuIs214 [myo-2p::GFP, unc-129p::itsn-1::GFP] III
HA3533	sod-1(tm776) II; unc-119(+) III; rtSi007 [sod-1p::sod-1H71Y^{M}::sod-1 3'UTR + Cbr-unc-119(+)] IV; nuIs214 [myo-2p::GFP, unc-129p::itsn-1::GFP] III
HA3534	sod-1(tm776) II; unc-119(+) III; rtSi006 [sod-1p::sod-1G85R^{M}::sod-1 3'UTR + Cbr-unc-119(+)] IV; nuIs214 [myo-2p::GFP, unc-129p::itsn-1::GFP] III
HA3535	sod-1(tm776) II; unc-119(+) III; rtSi026 [Cbr-unc-119(+)] IV; nuIs214 [myo-2p::GFP, unc-129p::itsn-1::GFP] III
GE24	pha-1(e2123) III
HA2986	sod-1(rt448[sod-1WT^{C}]) II; pha-1(+) III
HA2988	sod-1(rt450[sod-1L84V^{C}]) II; pha-1(+) III
HA3299	sod-1(rt451[sod-1G85R^{C}]) II; pha-1(+) III
HA2987	sod-1(rt449[sod-1G93A^{C}]) II; pha-1(+) III
HA3536	sod-1(+) II; [snb-1p::hSOD1WT::YFP]
HA3537	sod-1(tm776) II; [snb-1p::hSOD1WT::YFP]
HA3538	sod-1(rt448[sod-1WT^{C}]) II; pha-1(+) III; [snb-1p::hSOD1WT::YFP]
HA3539	sod-1(rt450[sod-1L84V^{C}]) II; pha-1(+) III; [snb-1p::hSOD1WT::YFP]
HA3540	sod-1(rt451[sod-1G85R^{C}]) II; pha-1(+) III; [snb-1p::hSOD1WT::YFP]
HA3541	sod-1(rt449[sod-1G93A^{C}]) II; pha-1(+) III; [snb-1p::hSOD1WT::YFP]
HA3542	sod-1(rt448[sod-1WT^{C}]) II; pha-1(+) III; vsIs48 [unc-17p::GFP]
HA3543	sod-1(rt450[sod-1L84V^{C}]) II; pha-1(+) III; vsIs48 [unc-17p::GFP]
HA3544	sod-1(rt451[sod-1G85R^{C}]) II; pha-1(+) III; vsIs48 [unc-17p::GFP]
HA3545	sod-1(rt449[sod-1G93A^{C}]) II; pha-1(+) III; vsIs48 [unc-17p::GFP]
HA3	sod-1(+) II; nuIs11 [osm-10p::GFP + lin-15(+)]
HA3546	sod-1(tm776) II; nuIs11 [osm-10p::GFP + lin-15(+)]
HA3547	sod-1(rt448[sod-1WT^{C}]) II; pha-1(+) III; nuIs11 [osm-10p::GFP + lin-15(+)]
HA3548	sod-1(rt451[sod-1G85R^{C}]) II; pha-1(+) III; nuIs11 [osm-10p::GFP + lin-15(+)]
OH7235	sod-1(+) II; zdIs13 [tph-1p::GFP] IV
HA3549	sod-1(tm776) II; zdIs13 [tph-1p::GFP] IV
HA3550	sod-1(rt448[sod-1WT^{C}]) II; pha-1(+) III; zdIs13 [tph-1p::GFP] IV
HA3551	sod-1(rt451[sod-1G85R^{C}]) II; pha-1(+) III; zdIs13 [tph-1p::GFP] IV
TG2435	sod-1(+) II; vtIs1 [dat-1p::GFP + rol-6(su1006)] V
HA3552	sod-1(tm776) II; vtIs1 [dat-1p::GFP + rol-6(su1006)] V
HA3553	sod-1(rt448[sod-1WT^{C}]) II; pha-1(+) III; vtIs1 [dat-1p::GFP + rol-6(su1006)] V
HA3554	sod-1(rt451[sod-1G85R^{C}]) II; pha-1(+) III; vtIs1 [dat-1p::GFP + rol-6(su1006)] V
