# Supplementary material for: Single copy/knock-in models of ALS SOD1 in C. elegans suggest loss and gain of function have different contributions to cholinergic and glutamatergic neurodegeneration
Source: PLoS Genet. 2018 Oct 8;14(10):e1007682. doi: 10.1371/journal.pgen.1007682 (PMC6200258; doi:10.1371/journal.pgen.1007682)
Supplement: S3 Table — (PDF) [file pgen.1007682.s007.pdf]

	H.s. **SOD1 amino acid location**	***C.e.*** **SOD-1 amino acid location**
A4V	5	5
H71Y	72	71
L84V	85	84
G85R	86	85
G93A	94	93
