# Supplementary material for: Single copy/knock-in models of ALS SOD1 in C. elegans suggest loss and gain of function have different contributions to cholinergic and glutamatergic neurodegeneration
Source: PLoS Genet. 2018 Oct 8;14(10):e1007682. doi: 10.1371/journal.pgen.1007682 (PMC6200258; doi:10.1371/journal.pgen.1007682)
Supplement: S4 Table — MST, Median Survival Time; n is the number of total subjects at the beginning of each trial and x is the total number of subjects censored by the end of the study; R stands for replica; Avg MST is the average of the three individual MST determinations; Pooled MST was calculated by pooling data from all replicates; SEM, standard error of the median; Und, undefined due to high number of censored subjects. In S1 Fig, we scored survival at 25 oC on alternating days from pre-adulthood (L4 stage) until death. In S2 Fig, we scored survival at 25 oC every day from pre-adulthood (L4 stage) in animals exposed to 2.5 mM paraquat. sod-1(+) is the standard N2 strain. Survival was scored in the absence of FUDR. Animals were transferred to new plates every other day to avoid progeny contamination. Animals that left the plate or animals with internal egg hatching were censored; these animals were included in lifespan determinations until the day before censoring. N was 30 for all genotypes in all panels in each of 3 independent trials, except for sod-1G85RC in “+” paraquat R1 trial which had 20 animals (S2B Fig). MST and log-rank test significance was determined using Graph Pad Prism 6 software (La Jolla, CA). (PDF) [file pgen.1007682.s008.pdf]

Panel	2.5 mM Paraquat	Genotype	R1		R2		R3		R1, R2, R3		Pooled		*P-value*					
			MST	n (x)	MST	n (x)	MST	n (x)	Avg MST	SEM	MST	n (x)	*vs sod-1(+)*	*vs sod-1WT*^{M}	*vs sod-1WT*^{C}	*vs hSOD1WT-YFP*	*vs sod-1(-); hSOD1WT-YFP*	*vs sod-1(-)*
	-	*sod-1WT*^{M}	11	30 (13)	17	30 (8)	13	30 (7)	13.67	1.76	13	90 (28)						
	-	*sod-1A4V*^{M}	11	30 (8)	13	30 (11)	7	30 (9)	10.33	1.76	11	90 (28)		<0.0001				
Fig S1A	-	*sod-1H71Y*^{M}	7	30 (5)	13	30 (7)	9	30 (8)	9.67	1.76	9	90 (20)		0.0051				
	-	*sod-1G85R*^{M}	13	30 (11)	13	30 (10)	11	30 (9)	12.33	0.67	13	90 (30)		0.6378				
	-	*empty*^{M}	9	30 (6)	11	30 (11)	9	30 (11)	9.67	0.67	9	90 (28)		0.0309				
	-	*sod-1(+)*	12	30 (19)	12	30 (4)	12	30 (7)	12.00	0.00	12	90 (30)						
	-	*sod-1WT*^{C}	14	30 (13)	14	30 (11)	14	30 (8)	14.00	0.00	14	90 (32)	0.8594					
Fig S1B	-	*sod-1L84V*^{C}	10	30 (12)	10	30 (5)	12	30 (8)	10.67	0.67	12	90 (25)			0.02			
	-	*sod-1G85R*^{C}	18	30 (7)	16	30 (7)	14	30 (10)	16.00	1.15	16	90 (24)			0.3181			
	-	*sod-1G93A*^{C}	10	30 (13)	8	30 (2)	12	30 (7)	10.00	1.15	12	90 (22)			0.0296			
	-	*sod-1(+)*	10	30 (24)	14	30 (17)	14	30 (10)	12.67	1.33	12	90 (51)						
Fig S1C	-	*hSOD1WT-YFP*^{OE}	10	30 (14)	14	30 (11)	16	30 (10)	13.33	1.76	14	90 (35)	0.3576					
	-	*hSOD1G85R-YFP*^{OE}	10	30 (13)	12	30 (12)	7	30 (4)	9.67	1.45	10	90 (29)				0.111		
	-	*sod-1(+)*	12	30 (19)	12	30 (4)	12	30 (7)	12.00	0.00	12	90 (30)						
Fig S1D	-	*sod-1(-)*	12	30 (6)	10	30 (3)	10	30 (4)	10.67	0.67	10	90 (13)	0.0418					
	-	*sod-1(-); hSOD1WT-YFP*^{OE}	12	30 (11)	10	30 (9)	14	30 (11)	12.00	1.15	12	90 (31)						0.442
	-	*sod-1(-); hSOD1G85R-YFP*^{OE}	16	30 (22)	14	30 (19)	16	30 (21)	15.33	0.67	16	90 (62)					0.0038	0.022
	+	*sod-1WT*^{M}	8	30 (23)	7	30 (29)	7	30 (12)	7.33	0.33	7	90 (64)						
	+	*sod-1A4V*^{M}	10	30 (29)	9	30 (26)	9	30 (20)	9.33	0.33	9	90 (75)		0.0001				
Fig S2A	+	*sod-1H71Y*^{M}	3	30 (20)	Und	30 (27)	3	30 (25)	3.00	0.00	3	90 (72)		<0.0001				
	+	*sod-1G85R*^{M}	3	30 (18)	Und	30 (21)	4	30 (27)	3.50	0.50	3	90 (66)		<0.0001				
	+	*empty*^{M}	3	30 (25)	3	30 (22)	4	30 (26)	3.33	0.33	3	90 (73)		<0.0001				
	+	*sod-1(+)*	Und	30 (28)	9	30 (27)	9	30 (19)	9.00	0.00	9	90 (74)						
	+	*sod-1(-)*	3	30 (19)	4	30 (24)	3	30 (24)	3.33	0.33	3	90 (67)	<0.0001					
Fig S2B	+	*sod-1WT*^{C}	9	30 (20)	7	30 (24)	Und	30 (28)	8.00	1.00	9	90 (72)	0.7472					
	+	*sod-1L84V*^{C}	9	30 (17)	9	30 (21)	5	30 (16)	7.67	1.33	8	90 (54)			0.0032			
	+	*sod-1G85R*^{C}	3	20 (7)	6	30 (18)	3	30 (20)	4.00	1.00	4	90 (45)			<0.0001			
	+	*sod-1G93A*^{C}	7	30 (15)	8	30 (22)	7	30 (22)	7.33	0.33	7	90 (59)			0.0071			
	+	*sod-1(+)*	7	30 (20)	7	30 (27)	6	30 (21)	6.67	0.33	7	90 (68)						
Fig S2C	+	*hSOD1WT-YFP*^{OE}	Und	30 (28)	6	30 (28)	6	30 (24)	6.00	0.00	6	90 (80)	0.0098					
	+	*hSOD1G85R-YFP*^{OE}	Und	30 (27)	2	30 (26)	3	30 (24)	2.50	0.50	3	90 (77)				<0.0001		
	+	*sod-1(+)*	9	30 (19)	9	30 (16)	9	30 (22)	9.00	0.00	9	90 (57)						
Fig S2D	+	*sod-1(-)*	3	30 (24)	3	30 (21)	Und	30 (30)	3.00	0.00	3	90 (75)	<0.0001					
	+	*sod-1(-); hSOD1WT-YFP*^{OE}	4	30 (27)	3	30 (23)	5	30 (25)	4.00	0.58	4	90 (75)						0.042
	+	*sod-1(-); hSOD1G85R-YFP*^{OE}	3	30 (13)	3	30 (7)	2	30 (9)	2.67	0.33	3	90 (29)					0.0002	0.0713
