# Supplementary material for: Single copy/knock-in models of ALS SOD1 in C. elegans suggest loss and gain of function have different contributions to cholinergic and glutamatergic neurodegeneration
Source: PLoS Genet. 2018 Oct 8;14(10):e1007682. doi: 10.1371/journal.pgen.1007682 (PMC6200258; doi:10.1371/journal.pgen.1007682)
Supplement: S5 Table — Directly comparable data sets are flanked by double lines. Appropriate controls are indicated within each experimental set. Genotypes with increases and decreases are indicated with a shade of red and blue, respectively. n.s., not significant; n.a., not available. (PDF) [file pgen.1007682.s009.pdf]

Panel	Assay	*sod-1(+)*	*sod-1(-)*	*sod-1WT*^{M}	*empty*^{M}	*sod-1A4V*^{M}	*sod-1H71Y*^{M}	*sod-1G85R*^{M}	*sod-1WT*^{C}	*sod-1L84V*^{C}	*sod-1G85R*^{C}	*sod-1G93A*^{C}	*hSOD1WT-YFP*^{OE}	*hSOD1G85R-YFP*^{OE}	*sod-1(-); hSOD1WT-YFP*^{OE}	*sod-1(-); hSOD1G85R-YFP*^{OE}
**Fig 2**	neuronal hSOD1 inclusions	control	n.s.	control	n.s.	increased	increased	increased	control	n.s.	increased	increased	same as in *sod-1(+)*	increased (Wang et al., 2009)	n.a.	n.a.
**Fig 3**	aldicarb sensitivity	control	increased	n.a.	increased	n.s.	increased	n.s.	control	n.s.	increased	n.s.	control	decreased; (Wang et al., 2009)	control	n.a.
**Fig 4**	PQ-induced cholinergic degeneration	control	n.s.	control	n.s.	increased	increased	increased	control	n.s.	increased	increased	control	increased	control	increased
**Fig 5**	PQ-induced glutamatergic degeneration	control	increased	control	increased	n.s.	increased	increased	control	increased	increased	n.s.	control	increased	control	increased
**Fig S1**	survival	control	decreased	control	decreased	decreased	decreased	n.s.	control	decreased	n.s.	decreased	control	n.s.	control	increased
**Fig S2**	PQ sensitivity	control	decreased	control	decreased	increased	decreased	decreased	control	decreased	decreased	decreased	control	decreased	control	decreased
